# Structure of HIV-1 Vpr in complex with the human nucleotide excision repair protein hHR23A

**DOI:** 10.1038/s41467-021-27009-w

**Published:** 2021-11-25

**Authors:** In-Ja L. Byeon, Guillermo Calero, Ying Wu, Chang H. Byeon, Jinwon Jung, Maria DeLucia, Xiaohong Zhou, Simon Weiss, Jinwoo Ahn, Caili Hao, Jacek Skowronski, Angela M. Gronenborn

**Affiliations:** 1 Pittsburgh Center for HIV Protein Interactions, University of Pittsburgh School of Medicine, Pittsburgh, PA USA; 2 Department of Structural Biology, University of Pittsburgh School of Medicine, Pittsburgh, PA USA; 3 Present Address: ABL Bio Inc., 16, Daewangpangyo-ro 712 beon-gil, Bundang-gu, Seongnam-si, Gyeonggi-do 13488 Republic of Korea; 4 Department of Molecular Biology and Microbiology, Case Western Reserve School of Medicine, Cleveland, OH USA

**Keywords:** X-ray crystallography, Virus-host interactions, Viral proteins, Solution-state NMR

## Abstract

HIV-1 Vpr is a prototypic member of a large family of structurally related lentiviral virulence factors that antagonize various aspects of innate antiviral immunity. It subverts host cell DNA repair and protein degradation machineries by binding and inhibiting specific post-replication repair enzymes, linking them via the DCAF1 substrate adaptor to the Cullin 4 RING E3 ligase (CRL4^DCAF1^). HIV-1 Vpr also binds to the multi-domain protein hHR23A, which interacts with the nucleotide excision repair protein XPC and shuttles ubiquitinated proteins to the proteasome. Here, we report the atomic resolution structure of Vpr in complex with the C-terminal half of hHR23A, containing the XPC-binding (XPCB) and ubiquitin-associated (UBA2) domains. The XPCB and UBA2 domains bind to different sides of Vpr’s 3-helix-bundle structure, with UBA2 interacting with the α2 and α3 helices of Vpr, while the XPCB domain contacts the opposite side of Vpr’s α3 helix. The structure as well as biochemical results reveal that hHR23A and DCAF1 use overlapping binding surfaces on Vpr, even though the two proteins exhibit entirely different three-dimensional structures. Our findings show that Vpr independently targets hHR23A- and DCAF1- dependent pathways and highlight HIV-1 Vpr as a versatile module that interferes with DNA repair and protein degradation pathways.

## Abstract (web summary)

Vpr is a HIV-1 accessory virulence factor that also interacts with the human DNA repair protein hHR23A. Here, the authors present the structure of Vpr in complex with the C-terminal half of hHR23A comprising the XPC-binding and ubiquitin-associated domains, which reveals that hHR23A interacts with the DCAF1-binding and not the substrate-binding Vpr surface and further illustrates how Vpr acts as a versatile structural adapter that targets diverse DNA repair pathways.

## Introduction

Vpr is one of the four HIV-1 accessory virulence factors, Vif, Vpr, Vpu, and Nef, that antagonize innate mechanisms and intrinsic cellular pathways that interfere with effective viral replication^1–7^. Closely structurally related orthologues and paralogues of HIV-1 Vpr are found in HIV-2 and essentially all primate lentiviruses^8–10^. While our knowledge of how Vpr promotes HIV-1 replication and pathogenesis is clearly incomplete, one well-established mechanism involves hijacking of the CRL4 E3 ubiquitin ligase and its DCAF1 substrate receptor (CRL4^DCAF1^) for depletion of cellular proteins that directly or indirectly target viral components. In particular, Vpr binds to DCAF1 and mediates loading of different protein substrates onto this E3 ligase, leading to their polyubiquitination and proteasome-dependent degradation^11–13^. Intriguingly, among these substrates are proteins involved in post-replication DNA repair and chromatin metabolism, including the base excision repair (BER) protein Uracil-DNA glycosylase (UNG2)^14,15^, the double strand break repair and Holliday junction resolvase MUS81-EME1^16,17^, the helicase-like transcription factor (HLTF) that plays a role in the repair of stalled replication forks^18–20^, Exo1, a nuclease involved in double strand break repair^21^, and TET2, a methylcytosine dioxygenase that is a potent epigenetic regulator of gene expression in hematopoietic cells^22^. The structural basis of E3 ligase targeting by Vpr was elucidated for the BER protein UNG2: the substrate receptor DCAF1 engages helix α3 and, on the opposing surface of Vpr, a loop between helices α2 and α3 inserts into the active site of UNG2, thereby interfering with DNA binding and catalysis^23^. Significantly, UNG2, Exo1, HLTF and TET2 were shown to interfere with HIV-1 infection, supporting the notion that Vpr antagonizes specific post-replication DNA repair proteins that restrict HIV-1, thereby facilitating HIV-1 replication^4,21,22,24^.

HIV-1 Vpr also binds tightly to hHR23A^25–27^, the human homolog of yeast Rad23, which not only partakes in nucleotide excision repair (NER) but also plays important roles in various other aspects of cellular metabolism, beyond DNA repair^28^. hHR23A and its hHR23B isoform are modular and comprise four independently folded domains, joined by three flexible linkers. In particular, they possess an N-terminal ubiquitin-like domain (UBL), a central ubiquitin-associated domain (UBA1), followed by a RAD4/XPC (xeroderma pigmentosum group C) binding domain (RBD/XPCB) and a C-terminal ubiquitin-associated domain (UBA2). hHR23 proteins participate in NER by forming a complex with the XPC protein, mediated by the XPCB domain, and play a role in the initial recognition of damaged sites in DNA^29^. In addition, the hHR23B–XPC complex was shown to function as a co-activator controlling particular transcription programs^30–32^. The UBL domain mediates 26S proteasome binding by engaging the Rpn1 and Rpn10 subunits^33,34^, while the UBA domains capture polyubiquitin-conjugated target proteins, especially Lys-48-conjugated chains^35^.

Previously, we and others demonstrated that Vpr, which comprises a three-helix bundle with flexible N- and C-terminal tails^23,36^, binds the C-terminal half of hHR23A, contacting the UBA2^25^ and the XPCB^37^ domains. Interestingly, in contrast to other DNA repair proteins that are targeted by Vpr, hHR23A does not become ubiquitinated in a Vpr-dependent manner and is not depleted in cells expressing HIV-1 Vpr^21^, suggesting that hHR23A is not a Vpr-recruited CRL4^DCAF1^ E3 substrate and that the Vpr–hHR23A complex likely presents a distinct architecture.

Here, we report the high-resolution NMR structure of Vpr, complexed with the C-terminal half of hHR23A, containing the XPCB and UBA2 domains, and a 2.2 Å resolution X-ray structure of the Vpr/UBA2 domain complex. Interestingly, our structural findings reveal that hHR23A interacts with the DCAF1-binding and not the substrate-binding Vpr surface, unlike other previously characterized Vpr targets involved in post-replication DNA repair pathways, and that this interaction antagonizes DCAF1 binding. We also show that Vpr binds hHR23A in a complex with XPC. Thus, our findings establish that HIV-1 Vpr interfaces with DNA repair pathways not only via CRL4^DCAF1^ E3 and proteasomal degradation of targets, but also through the hHR23/XPC complex, suggesting the possibility of an alternative to the ubiquitin ligase route. Our findings further illustrate how HIV-1 Vpr acts as a versatile structural adapter that targets diverse DNA repair pathways.

## Results

### Vpr/hHR23A complex preparation and NMR and X-ray studies

Previous work from our laboratory demonstrated that the C-terminal half of hHR23A, hHR23A_223–363_, comprising both XPCB and UBA2 domains, directly binds Vpr^37^. Although the Vpr_1–79_/hHR23A_223–363_ complex could be prepared after co-expression, purification in amounts for structural studies proved challenging, and screening by ^1^H–^15^N HSQC NMR spectroscopy revealed a mixture of complex and free hHR23A_223–363_ proteins^37^. Therefore, in order to prepare a homogeneous sample of the complex, we generated Vpr_1–79_ and hHR23A_223–363_ fusion constructs, connecting Vpr to either the N- or C-terminus of the double domain construct of hHR23A by a six amino acid (GGS)_2_ linker (L) (details are provided in the “Methods” section). Assessment of the structural properties of the linked proteins was performed by ^1^H–^15^N HSQC spectroscopy. While the spectrum of the C-terminal fusion construct, hHR23A_223–363_–L-Vpr_1–79_, exhibited severe NMR line-broadening and loss of ^1^H–^15^N HSQC cross peaks (Supplementary Fig. 1a) compared to the free hHR23a protein, the spectrum of the N-terminal fusion construct, Vpr_1–79_–L-hHR23A_223–363_ (Supplementary Fig. 1b), was of sufficient quality to proceed with resonance assignments and structure determination. This suggested that the six amino acid containing linker at the end of Vpr_1–79_ was long enough to allow for correct positioning of the two proteins in the complex, while it was most likely too short to permit correct complex formation when used to connect the C-terminus of hHR23A to the N-terminus of Vpr. Good agreement for the amide resonances in the ^1^H–^15^N HSQC spectrum of the Vpr_1–79_–L-hHR23A_223–363_ linked and the Vpr_1–79_ and hHR23A_223–363_ co-expressed complexes are noted (Supplementary Fig. 1c), confirming that essentially identical complexes are formed by the linked and unlinked proteins.

**Fig. 1:**
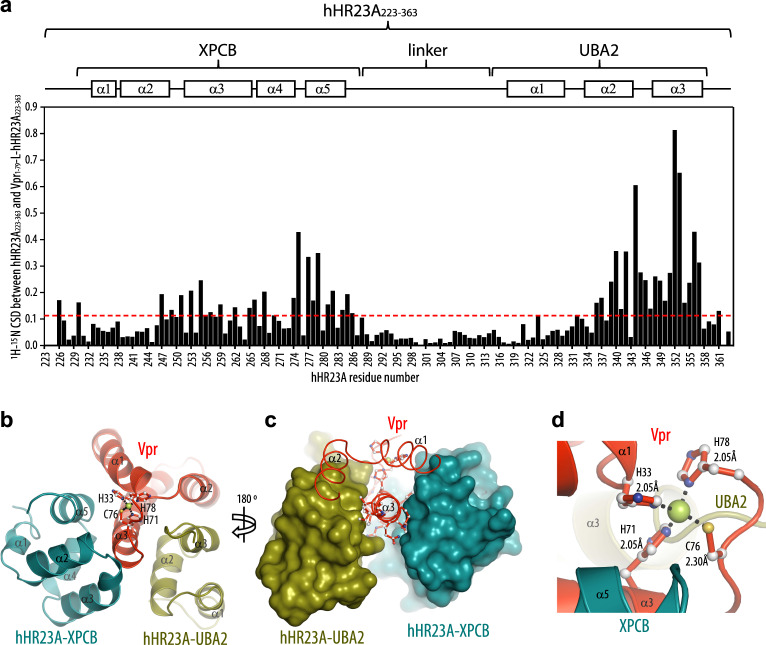
Interaction of hHR23A and Vpr in the Vpr_1–79_–L-hHR23A_223–363_ complex. **a** Mapping of the Vpr binding site on hHR23A_223–363_ in the Vpr_1–79_–L-hHR23A_223–363_ complex by NMR. The ^1^H,^15^N-combined chemical shift differences (CSDs), calculated using the equation, (Δ*δ*_HN_^2^ + (Δ*δ*_N_/6)^2^)^1/2^ (for all amino acids, but prolines) or |Δ*δ*_Hα_| (for prolines), with Δ*δ*_HN_, Δ*δ*_N_ and Δ*δ*_Hα_ representing the ^1^HN, ^15^N and H_α_ chemical shift differences between resonance frequencies of free hHR23A_223–363_ and those in the Vpr_1–79_–L-hHR23A_223–363_ complex, are plotted along the linear amino acid sequence. The average CSD value is drawn in a red dotted line. Domain and secondary structure elements are schematically indicated at the top. **b**, **c** Lowest energy NMR conformer of the Vpr_1–79_–L-hHR23A_223–363_ complex in ribbon representation (**b**) and worm (Vpr_1–79_) and space filling representation (hHR23A_223–363_) (**c**). **d** Close-up view of the Zn^2+^-binding motif. Distances between the Zn^2+^ ion and His and Cys atoms are labeled. Helices are labeled as α1, α2 and α3.

NMR assignments for the linked Vpr_1–79_–L-hHR23A_223–363_ construct, in which Vpr is intramolecularly bound to hHR23A_223–363_, were obtained for 97% of the backbone resonances and 92% of all side chain resonances. Chemical shift-derived secondary structure elements for Vpr and the XPCB and UBA2 domains (Supplementary Fig. 1d) are essentially the same as those for free Vpr^36^ and the individual hHR23A domains^38–40^. The amide resonances for the 13 N-terminal Vpr residues, those of the (GGS)_2_ linker, the first seven residues (223–229) before XPCB, the flexible linker residues (288–314) between XPCB and UBA2 and the last residues (360–363) following UBA2 are sharp and exhibit only small secondary shifts, compared to random coil values (Supplementary Fig. 1d), indicating that these regions are mobile and essentially adopt random coil conformations. Comparison between assignments for the Vpr-bound portion of hHR23A in Vpr_1–79_–L-hHR23A_223–363_ and free hHR23A_223–363_ permitted unequivocal delineation of the Vpr binding site on hHR23A by chemical shift mapping. This refined and completed the details of our earlier binding site determination^37^, which for the non-linked Vpr-complexed hHR23A had been largely based on comparing amide backbone assignments. We observed significant chemical shift differences (CSDs) (>0.2 ppm) for resonances associated with all helices, but α1, in the XPCB domain, confirming that most of XPCB is involved in binding. Even larger CSDs (>0.3 ppm) were observed for amide resonances belonging to the UBA2 domain, in particular for resonances associated with helices α2 and α3. Resonances of amino acids in the first helices of both, the XPCB and UBA2 domains, exhibited only very small CSDs, indicating that they are marginally affected by the interaction with Vpr. The magnitude and organization of all CSDs along the hHR23A_223–363_ amino acid sequence are summarized in Fig. 1a.

Crystallization of Vpr_1–79_–L-hHR23A_223–363_ yielded crystals belonging to the P3_1_21 space group. Surprisingly, no electron density could be located for the XPCB domain, most likely resulting from its loss due to proteolytic cleavage during crystallization. Mass spectrometry of the crystals confirmed the presence of the UBA2 domain but provided no evidence for the presence of the XPCB domain (Supplementary Fig. 2). Crystals contained four Vpr and two hHR23A-UBA2 molecules in the asymmetric unit, and the structure was solved using the phases from the native Vpr Zn^2+^ anomalous signal, collected at the Zn^2+^ edge (9.65 keV) to 3.0 Å resolution and further extended to 2.2 Å from a dataset collected at the Se edge (12.65 keV). The model was refined using the program BUSTER^41,42^, followed by manual model building in Coot^43^, to a final *R*_work_/*R*_free_ value of 19.7/21.6%, respectively (see Table 1).

**Table 1:** Data collection and refinement statistics.

	Vpr_1–79_/hHR23A-UBA2 (Native)^a^	Vpr_1–79_/hHR23A-UBA2 (Zinc)^b^
*Data collection*		
Space group	P3_1_21	P3_1_21
*Cell dimensions*		
*a*, *b*, *c* (Å)	130.15, 130.15, 81.31	129.48, 129.48, 80.77
*α*, *β*, *γ* (°)	90.00, 90.00, 120.00	90.00, 90.00, 120.00
Resolution (Å)	50–2.29	50–3.10
*R*_merge_	8.6 (247.2)	19.5 (387.1)
*R*_sym_	7.7 (214.4)	18.9 (387.1)
I/*σ* (I)	9.1 (0.7)	12.8 (0.7)
CC_1/2_	99.9 (23.0)	99.9 (22.6)
Completeness (%)	90.6 (86.3)	99.9 (98.7)
Redundancy	3.5 (2.7)	18.0 (13.6)
*Refinement*		
FOM (SAD)^c^		0.37
Resolution (Å)	40–2.29	40–3.0
No. of reflections	29,904	15,409
*R*_work_/*R*_free_	19.7/21.6	21.3/23.7
No. of atoms of protein	2841	2841
*B* factors (Å^2^)	34	50
*R.M.S. deviations*		
Bond lengths (Å)	0.05	0.08
Bond angles (°)	1.1	0.93

Numbers in parentheses correspond to the highest resolution shell (2.43–2.29 and 3.18–3.10 Å, respectively).

^a^Data set was recorded at a wavelength of 0.97946 Å.

^b^Data set was recorded at a wavelength of 1.27368 Å.

^c^Phasing was performed using the program Crank (CCP4i suite)

### Overall structure of the Vpr/hHR23A complex

The NMR solution structure of the linked Vpr_1-79_–L-hHR23A_223–363_ complex (Fig. 1b–d) was calculated on the basis of 7650 NOE-derived distance constraints, including 81 Vpr/XPCB and 257 Vpr/UBA2 intermolecular distance constraints, and 330 dihedral angle constraints. The final 55-conformer ensemble of the complex satisfies all experimental constraints and displays excellent covalent geometry with atomic root-mean-square differences (r.m.s.d.) of 0.67 ± 0.06 and 1.13 ± 0.05 Å for the backbone and all heavy atoms, respectively, relative to their mean position (Table 2 and Supplementary Fig. 1e). The Vpr structure consists of three anti-parallel α-helices packed into a helical bundle, and very few conformational changes are noted in the hHR23A-bound Vpr, compared to the free structure^36^. Vpr’s helix α3 is buried between the XPCB and UBA2 domains (Fig. 1b, c). Further interactions in the complex involve Vpr’s α2 and UBA2’s α3 helices. In the X-ray structure of the Vpr/UBA2 domain complex, the features of the interaction interface are very similar to those observed in the NMR structure: the backbone r.m.s.d. between the NMR and X-ray structures for residues 14–78 of Vpr and residues 315–359 of UBA2 is 1.07 Å (Supplementary Fig. 1f).

**Table 2:** Statistics for the final 55 conformer ensemble of the Vpr_1-79_–L-hHR23A_223–363_ complex.

*Number of NOE distance constraints*	
Intra-residue (*i* − *j* = 0)	2377
Sequential (*i* – *j* = 1)	1806
Medium range (2 ≤ *i* − *j* ≤ 4)	1972
Vpr long range (*i* − *j* ≥ 5)	327
hHR23A long range (*i* − *j* ≥ 5)	830
Vpr–XPCB intermolecular	81
Vpr–UBA2 intermolecular	257
Total	7650
Number of dihedral angle constraints	
ϕ	166
ψ	164
Total	330
*Structural quality*	
Violations^a^	
Distance constraints (Å)	0.009 ± 0.001
Dihedral angle constraints (deg)	0.276 ± 0.056
*Deviation from idealized covalent geometry*	
Bond lengths (Å)	0.001 ± 0.000
Bond angles (deg)	0.385 ± 0.003
Improper torsions (deg)	0.216 ± 0.006
Average root-mean-square deviation of atomic	
coordinates (Å)^b,c^	
Backbone heavy atoms	0.67 ± 0.06
All heavy atoms	1.13 ± 0.05
*Ramachandran analysis (%)*^c^	
Most favorable regions	88.8 ± 1.8
Additional allowed regions	10.5 ± 1.9
Generously allowed regions	0.7 ± 0.5
Disallowed regions	0 ± 0.1

^a^No individual member of the ensemble exhibited distance violations >0.5 Å or dihedral angle violations >5°.

^b^Calculated for Vpr residues 14–78 and hHR23A residues 230–287 (XPCB) and 315–359 (UBA2) of individual structures with respect to the mean structure.

^c^Flexible regions (Vpr residues 1–13 and 79, the linker residues GGSGGS, the hHR23A residues 223–229, 288–314 and 360–363, and the C-terminal His_6_ tag) were excluded from the statistics.

The X-ray structure of Vpr in complex with the UBA2 domain revealed atomic details of Vpr’s Zn^2+ -^binding motif, which had not been resolved previously in either the X-ray structure of the DDB1/DCAF1/Vpr/UNG2 complex^23^ (PDB: 5JK7) or in the NMR structure of free Vpr^36^. The Zn^2+^ atom is coordinated by residues His33, His71, Cys76 and His78 at the C-terminal end of Vpr’s helix α3 (Fig. 1d and Supplementary Fig. 1f). His78 is the penultimate amino acid in the current Vpr construct and the Zn-binding cluster is important for maintaining the global and local architecture of Vpr.

### Vpr/UBA2 domain binding interface

**Fig. 2:**
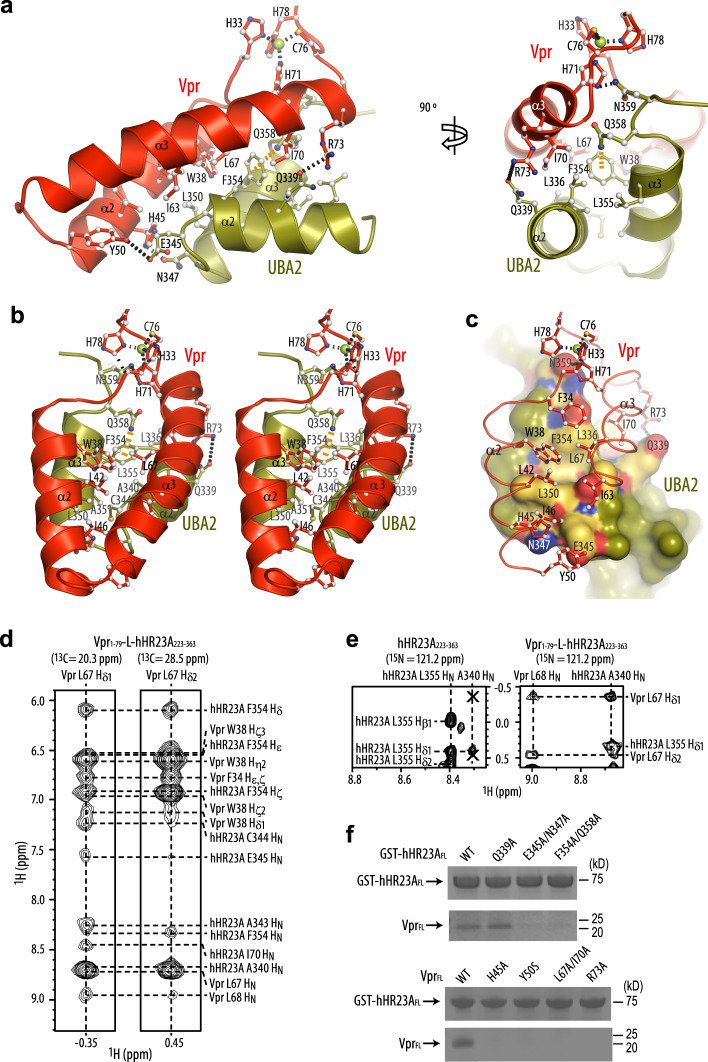
The Vpr and UBA2 interface in the Vpr_1-79_–L-hHR23A_223–363_ complex. **a** Interactions between the α2 and α3 helices of Vpr_1–79_ and the α2 and α3 helices in the UBA2 domain in two orientations, **b** stereoview with Vpr_1–79_ at the front. The Vpr α1 and UBA2 α1 helices that are not involved in the Vpr/UBA2 interface are omitted for clarity. In **a** and **b** Vpr and UBA2 are shown in red and olive ribbon representation, respectively, with selected sidechains in ball-and-stick representation. **c** worm (Vpr) and surface (UBA2) representations, with the Vpr α1 helix and residues 51–62 omitted for clarity. Vpr is shown in red and UBA2 in olive. Selected H-bonds and the Zn-coordinating bonds are indicated by dotted black lines and the NH_2_–pi H-bond in orange. Interacting residues in UBA2 are labeled and colored in gold (hydrophobic), red (negatively charged) and blue (positively charged). **d** Selected regions of the 3D ^13^C-edited NOESY spectrum of the Vpr_1–79_–L-hHR23A_223–363_ complex, illustrating NOEs involving Vpr L67 H_δ_ methyl protons. **e** Selected regions of the 3D ^15^N-edited NOESY spectra of free hHR23A_223–363_ (left) and the Vpr_1–79_–L-hHR23A_223–363_ complex (right), illustrating intra-hHR23A_223–363_ NOEs and inter-molecular NOEs between hHR23A_223–363_ and Vpr_1–79_ for the A340 H_N_ proton. The missing intermolecular cross-peaks in the free hHR23A_223–363_ spectrum are indicated by the symbols X. **f** GST-pull down data for WT and mutant hHR23A_FL_ proteins (top) and Vpr_FL_ (bottom). Source data are provided as a Source Data file. Three independent experiments were performed yielding similar results.

The binding interfaces on both Vpr and the UBA2 domain are extensive, burying a surface area of 760 Å^2^. Amino acids F34, W38, L42, I46, I63, L67 and I70 (helices α2 and α3) of Vpr pack against UBA2 residues F354, L350, A343, A340, Q339 and L336 (helices α3 and α2) in an antiparallel manner (Fig. 2a–c and Supplementary Fig. 3a–c). In addition, residues at the C- and N-terminal ends of Vpr’s α2 and α3 helices, H45, Y50, R73 and H71 and UBA2′s α2 and α3 helices, Q339, E345, N347 and N359, engage in hydrogen bonding and ionic interactions that contribute to binding (Fig. 2a). Amino acids in the Vpr–UBA2 intermolecular interface are listed in Supplementary Table 1. Within the UBA2 domain, an intramolecular NH···pi H-bond between the Q358 N_ε2_H_2_ amino group and the F354 aromatic ring is seen in the structure (Fig. 2a, b, orange dashed line), evidenced by the presence of intramolecular NOEs (Supplementary Fig. 3e) and a very large ring current-induced upfield shift of the Q358 amino group hydrogens (6.30, 6.46 ppm). The F354 aromatic ring is inserted into a small hydrophobic pocket lined by the side chains of Vpr’s F34, W38 and L67 (Fig. 2a–c). These binding interfaces in the Vpr_1–79_–L-hHR23A_223–363_ complex are clearly delineated by intermolecular NOEs (Fig. 2d, e, right and Supplementary Fig. 3e, f). The 3D NOESY data of the Vpr_1–79_–L-hHR23A_223–363_ complex were cross-checked and confirmed by comparison with the 3D NOESY data recorded on a sample of free hHR23A_223–363_ (Fig. 2e, left). As illustrated, intermolecular NOEs between the A340 H_N_ of hHR23A and L67 H_δ_ of Vpr are only present in the spectrum of the Vpr_1–79_–L-hHR23A_223–363_ complex (Fig. 2e, right) and not in that of the free hHR23A_223–363_ (shown by two X symbols in Fig. 2e, left).

The Vpr/UBA2 interface was validated by mutagenesis of the involved amino acids on both hHR23A_FL_ and Vpr_FL_. Several mutants of hHR23A_FL_ were examined: the E345A/N347A mutant was tested, since E345 and N347 form a salt bridge and a hydrogen bond with Vpr Y50 and H45, respectively (Supplementary Fig. 3a); the F354A/Q358A mutant was assessed since the aromatic side chain is integral to the UBA2 structure and thus it was expected that this mutation would compromise the integrity of the fold (Fig. 2a–c and Supplementary Fig. 3b); the single Q339A mutant was tested, since Q339 forms a salt bridge with R73 of Vpr at the periphery of the Vpr–UBA2 interface (Fig. 2a–c and Supplementary Fig. 3c). The two double mutants, E345A/N347A and F354A/Q358A, abolished Vpr binding (Fig. 2f, top panel), while the single mutant Q339A had little effect. On the Vpr side, H45A, Y50S, R73A and the double mutant L67A/I70A were tested. All of these Vpr_FL_ mutants exhibited impaired binding to hHR23A_FL_ (Fig. 2f, bottom panel). Thus, all these residues are implicated in structural integrity or binding and changing them either destabilizes Vpr’s fold or removes important contacts, thereby indirectly or directly interfering with binding.

Previous work in our^37^ and other^25^ laboratories showed that hHR23A’s UBA2 domain, but not the UBA1 domain, is involved in Vpr binding. Indeed, the structure presented here and our site-directed mutagenesis experiments validate this model. Most of the UBA2 residues that are important for the interaction with Vpr (Fig. 2a–c) are not present in the UBA1 domain, such as L336 (R179 in UBA1), L350 (R193 in UBA1), F354 (Y197 in UBA1), Q339 (A182 in UBA1), E345 (Y188 in UBA1), Q358 (G201 in UBA1) and N359 (I202 in UBA1) (Supplementary Fig. 3d).

### Vpr/XPCB domain binding interface

**Fig. 3:**
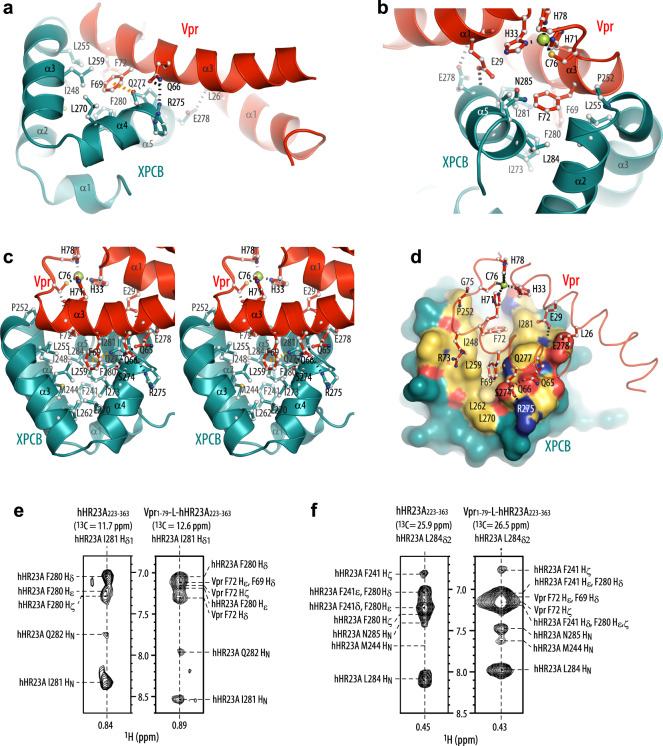
The Vpr and XPCB interface in the Vpr_1–79_–L-hHR23A_223–363_ complex. **a**, **b** Interactions between Vpr_1–79_ and the XPCB domain in the Vpr_1–79_–L-hHR23A_223–363_ complex, highlighting residues F69 and F72 in Vpr. **c** stereoview in a different orientation. In **a**–**c** Vpr and XPCB are shown in red and teal ribbon representation, respectively, with selected sidechains in ball-and-stick representation. **d** worm (Vpr) and surface (XPCB) representations. Vpr is shown in red and the XPCB domain in teal. Selected amino acids are shown in ball-and-stick representation (Vpr), and H-bonds and the Zn-coordinating bonds are indicated by dotted black lines and the NH_2_–pi H-bond in orange. Interacting residues in the XPCB domain are labeled and colored in gold (hydrophobic), red (negatively charged) and blue (positively charged). **e**, **f** Selected regions of the 3D ^13^C-edited NOESY spectra of free hHR23A_223–363_ (left) and the Vpr_1–79_–L-hHR23A_223–363_ complex (right), illustrating intra-hHR23A_223–363_ NOEs and inter-molecular NOEs between hHR23A_223–363_ and Vpr_1–79_ for I281 H_δ_ (**e**) and L284 H_δ_ (**f**) methyl protons.

The XPCB domain interacts with the α1 and α3 face of Vpr (Fig. 1b, c), burying a surface area of 670 Å^2^, somewhat smaller than the UBA2–Vpr interface. The binding interface between Vpr and the XPCB domain (Fig. 3a–d) in the NMR structure of Vpr_1–79_–L-hHR23A_223–363_ was identified by several intermolecular NOEs in the 3D NOESY spectrum (Fig. 3e, f, right and Supplementary Fig. 3g), cross-checked against NOEs present in the 3D NOESY data of free hHR23A_223–363_ (Fig. 3e, f, left) where the intermolecular NOEs between the hHR23A I281_Hδ1_/L284_Hδ2_ and the Vpr F72/F69 aromatic resonances are missing. The main contacts involve the phenyl rings of F69 and F72 of Vpr that insert into a hydrophobic cleft on XPCB (Fig. 3a–d). In the current structure, Vpr F69 is surrounded by a cluster of XPCB hydrophobic residues that include I248, L255, L259, L270, I273, F280, I281 and L284. The Vpr F72 side chain is bordered by XPCB I248, P252, L255, F280, I281 and L284. In addition, an intermolecular NH···pi interaction is present between a side-chain N_ε2_H_2_ proton of XPCB’s Q277 and the phenyl ring of Vpr’s F69 (Fig. 3a, c, orange dashed line), consistent with the observation of the intermolecular NOEs (Supplementary Fig. 3g) and the large ring current-induced upfield shift of one of the Q277 N_ε2_H_2_ protons from the free positions of 7.36 and 6.55 ppm to 7.01 and 6.19 ppm. Several other H-bonds between Vpr and the XPCB domain are also observed: E29–E278, Q66–R275 and Q66–S274 (Fig. 3a–d). All residues directly involved in the Vpr–XPCB intermolecular interface, as evidenced by inter-molecular NOEs, are summarized in Supplementary Table 2. Notably, F69 is a key Vpr residue for DCAF1 binding^23^, suggesting that DCAF1 and XPCB binding is mutually exclusive (see also below).

### HIV-1 Vpr binds both the hHR23A and hHR23B isoforms

**Fig. 4:**
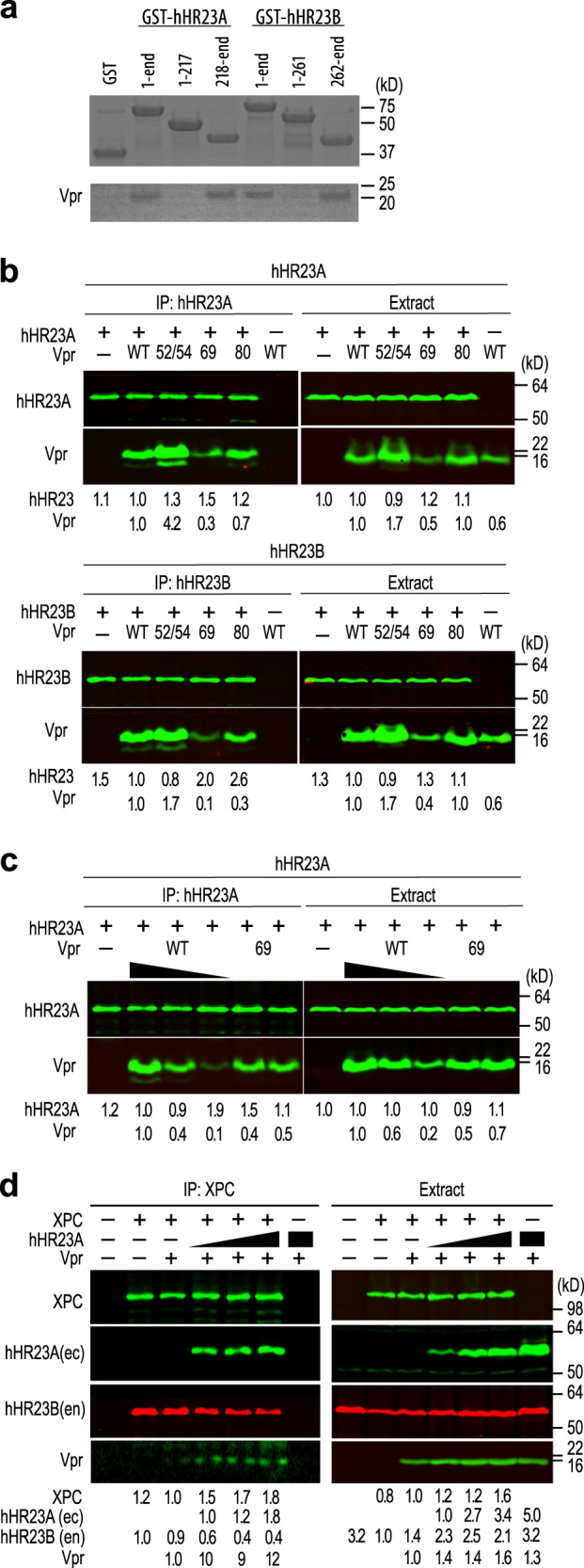
Vpr binds both the hHR23A and hHR23B isoforms and the hHR23/XPC DNA recognition complex. **a** Recombinant GST-hHR23A, residues 1–363, 1–217 and 218–363 and GST-hHR23B, residues 1–409, 1–261 and 262–409 were used in GST-pull down experiments with recombinant Vpr_FL_. Three replicates were performed for each experiment yielding similar results. **b** Vpr mutant binding to hHR23A (top) and hHR23B (bottom). FLAG-tagged hHR23A_FL_ (top) or hHR23B_FL_ (bottom) complexes were immunoprecipitated, via FLAG-tag, from HEK293T cell extracts transiently co-expressing the respective hHR23 isoform and HA-tagged Vpr_FL_ wild type (WT) or with the following mutations: D52R/W54R (52/54), F69A (69), R80A (80). Cell extracts and hHR23 immune complexes were analyzed by immunoblotting with antibodies specific for the FLAG and HA epitope tags, to detect the ectopically expressed FLAG-hHR23A, FLAG-hHR23B and HA-Vpr proteins. **c** Vpr F69A mutant binding to hHR23A. Expression levels of the HA-Vpr (F69A) and Vpr WT were varied by transfecting different amounts of plasmids expressing these proteins and their binding to FLAG-hHR23A was assessed as described above. **d** Vpr binds the hHR23/XPC complex. FLAG-tagged XPC, HA-tagged hHR23A_FL_ and myc-tagged Vpr_FL_ were expressed alone or in combinations in HEK293 T cells. Cell extracts and XPC immune complexes were analyzed by immunoblotting as described above except that the endogenously expressed hHR23B(en) protein was detected with an antibody specific for the hHR23B isoform. Immune complexes were revealed with fluorescent secondary antibodies and fluorescent signals quantified using a Li-Cor Odyssey imager. Relative levels of the indicated proteins in cell extracts and immune-complexes are shown. **b**–**d** Two biological replicates were performed for each experiment yielding similar results. The biological replicate of the experiment shown in panel **d** is shown in Supplementary Fig. 4. **a**–**d** Source data are provided as a Source Data file.

Most studies concerning NER have focused on hHR23B, rather than hHR23A. Given that the XPCB and UBA2 domains of hHR23A and hHR23B exhibit very high amino acid sequence identity (89%), we hypothesized that Vpr also interacts with hHR23B and investigated this possibility using in vitro and in vivo assays. Indeed, GST pull-down experiments indicated an interaction between Vpr and hHR23B, with the binding region localized to the C-terminal half of hHR23B (Fig. 4a). In addition, immunoprecipitation experiments revealed the presence of Vpr in both hHR23A (Fig. 4b, top) and hHR23B (Fig. 4b, bottom) immune complexes isolated from HEK293T cells co-expressing either hHR23 isoform together with Vpr, confirming that Vpr can form a complex with either hHR23 isoform in vivo. We also tested the effect of selected Vpr amino acid changes for binding hHR23A and hHR23B. Significantly, none of the variants tested, including the D52R/W54R one in which key interactions with the DNA repair protein UNG2 were abrogated, or the Vpr R80A variant, which no longer is able to activate G2 DNA damage checkpoint^44^, abolished binding to either hHR23 isoform, consistent with the results of our structural analyses of Vpr–hHR23A complex. In contrast, the Vpr F69A substitution, which disrupts Vpr binding to DCAF1^23^, resulted in a reduced Vpr protein steady state level in cell extracts and binding to hHR23 (Fig. 4b). To ascertain whether the latter was simply due to the lower level of Vpr F69A protein in cells, or whether the F69 side chain is indeed critical for hHR23 binding, dose response binding experiments were performed. They were conducted under conditions where Vpr WT and the F69A variant were present at similar levels. Interestingly, similar amounts co-precipitated with hHR23A (Fig. 4c). This suggests that F69 is not critical for hHR23 binding, and that contacts made by other residues, including Q66 and F72, with the XPCB domain, as well as contacts by W38, L67 and I70 with the UBA2 domain are sufficient to stabilize the complex.

### HIV-1 Vpr binds the hHR23A/XPC complex

hHR23 binds XPC, and this complex functions in DNA repair. Independently, hHR23 is also involved in ubiquitin-mediated proteolytic pathways. The latter does not require its association with XPC. To assess whether hHR23 has the potential to connect Vpr to NER, we asked whether Vpr can bind hHR23 in complex with XPC. To this end, XPC was co-expressed with Vpr in the absence or presence of increasing amounts of hHR23A, and XPC immune complexes were analyzed by immunoblotting for XPC, ectopically expressed hHR23A, endogenously expressed hHR23B isoform, and Vpr.

As shown in Fig. 4d and Supplementary Fig. 4, ectopic hHR23A expression resulted in increased amounts of Vpr co-precipitating with XPC. The low baseline levels of Vpr found in XPC immune complexes in the absence of ectopically expressed hHR23A probably reflect Vpr recruitment into the XPC complex by endogenously expressed hHR23A(B) isoforms, since the endogenous hHR23B is readily detectable in the XPC immune complexes. These findings support the model in which hHR23A(B) connects Vpr to hHR23A(B)/XPC mediated functions but does not exclude its involvement with XPC-independent hHR23 functions.

### Vpr interactions with hHR23A and DCAF1 are mutually exclusive

**Fig. 5:**
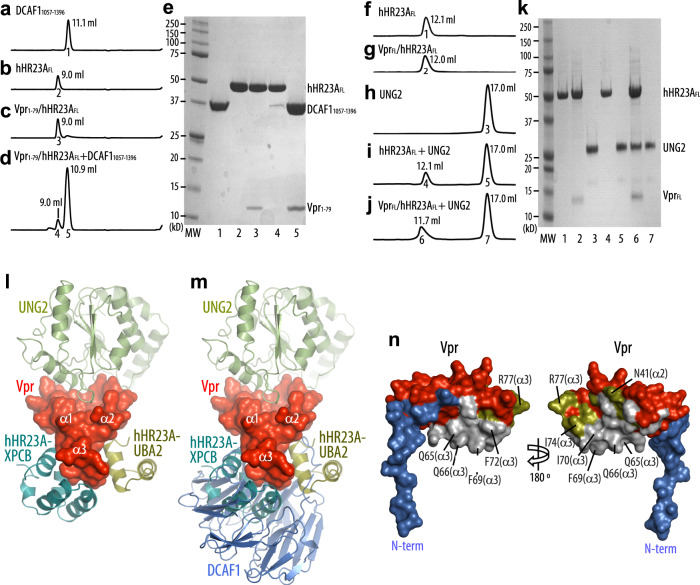
hHR23A is displaced from the Vpr/hHR23A complex by DCAF1. Analytical gel-filtration chromatography elution profiles of **a** DCAF1_1057–1396_, **b** hHR23A_FL_, **c** the Vpr_1–79_/hHR23A_FL_ complex, **d** an equimolar mixture of the Vpr_1–79_/hHR23A_FL_ complex and DCAF1_1057–1396_ and **e** SDS–PAGE analysis of the elution peaks; Lane numbers correspond to peak numbers in (**a**–**d**). Analytical gel-filtration chromatography elution profiles of **f** hHR23A_FL_, **g** the Vpr_FL_/hHR23A_FL_ complex, **h** UNG2, **i** an equimolar mixture of hHR23A_FL_ and UNG2, **j** an equimolar mixture of the Vpr_FL_/hHR23A_FL_ complex and UNG2 and **k** SDS–PAGE analysis of the elution peaks. Lane numbers correspond to peak numbers in (**f**–**j**). Two independent experiments were performed yielding similar results. **l**, **m** Molecular model of the UNG2/Vpr_1–79_/hHR23A_223–363_ complex based on the superposition of the UNG2/Vpr_1–79_/DCAF1_1045–1396_ complex and the current Vpr_1–79_–L-hHR23A_223–363_ complex without (**l**) and with displaying DCAF1 (**m**). **n** Surface representation of Vpr_1–79_ in two views, highlighting residues with DCAF1_1045–1396_ contacts (blue), with both DCAF1_1045–1396_ and hHR23A_223–363_ contacts (silver), and with hHR23A_223–363_ UBA2 only contacts (olive). Vpr_1–79_ residues that do not interact with DCAF1_1045–1396_ or hHR23A_223–363_ are shown in red.

Previous genetic and biochemical studies linked several cellular effects of Vpr to its binding to DCAF1 and, by extension, to the subversion of CRL4^DCAF1^ E3. Interestingly, comparison between the Vpr_1–79_–L-hHR23A_223–363_ (this study) and the UNG2/Vpr_1–79_/DCAF1_1045–1396_/DDB1 (PDB: 5JK7) structures reveals that both DCAF1 and hHR23A interact with the α3 helix of Vpr (Fig. 5l–n and Supplementary Fig. 5a, b). However, engaging these different binding partners results in only a small change in the Vpr structure, namely a slight shift in the Vpr α3 helix (Supplementary Fig. 1g). The buried surface area in the Vpr_1–79_/DCAF1_1045–1396_ region is slightly larger than in the Vpr_1–79_–L-hHR23A_223–363_ complex (1670 vs. 1430 Å^2^, respectively) and removal of DCAF1 residues 1045–1056 permitted purification of DCAF1 without DDB1^15^, consistent with previous findings about DDB1 binding^23^. Therefore, our data suggest that Vpr interactions with hHR23A and DCAF1 are competitive, with binding to DCAF1 likely of higher affinity. We hence tested whether DCAF1_1057–1396_ can compete and displace hHR23A from Vpr_1–79_ (Fig. 5a–e). When using DCAF1_1057–1396_ in the presence of a 1:1 (M/M) mixture of Vpr_1–79_ and hHR23A_FL_ in size exclusion column chromatography, an elution peak for free hHR23A_FL_ (Fig. 5d, peak 4; Fig. 5e, lane 4) and a peak for the Vpr_1–79_/DCAF1_1057–1396_ complex (Fig. 5d, peak 5; Fig. 5e, lane 5) is observed, confirming that Vpr binds DCAF1_1057–1396_ more tightly than hHR23A_FL_. This again confirms that both these Vpr-binding proteins engage the same surface on Vpr for their interaction, namely helix α3, as structurally confirmed by the best-fit superposition of the Vpr_1–79_–L-hHR23A_223–363_ and the Vpr_1–79_/DCAF1_1045–1396_ complex structures (Fig. 5l–n and Supplementary Fig. 5a, b). Both hHR23A_223–363_ and DCAF1_1045–1396_ utilize hydrophobic residues to contact F69, I70 and F72 and charged residues to form hydrogen bonds with Q65 and Q66 (Fig. 5n). Non-shared interactions include residues from helices α2 and α3 (N41, I74 and R77), which bind the UBA2 domain, and the Vpr N-terminus that wraps around the side of DCAF1’s WD40 motif (Fig. 5n). The higher Vpr-binding affinity of DCAF1 was further confirmed by isothermal titration calorimetry (ITC): DCAF1 exhibited a *K*_d_ of 116 ± 47 nM while the hHR23A *K*_d_ was 640 ± 408 nM (Supplementary Fig. 5c, d). The above findings clearly demonstrate that Vpr binding to DCAF1 and hHR23 is mutually exclusive and suggest that hHR23 provides an alternative to the DCAF1 (CRL4^DCAF1^ E3)-mediated mechanisms for Vpr to exert its function.

The hHR23A_223–363_ interaction with Vpr_1–79_ spans two of the three surfaces created by Vpr’s three helix bundle (Fig. 1b, c), permitting the third surface, particularly Vpr helices α1 and α2, to potentially engage in additional interactions. Previous work by our laboratory demonstrated that UNG2 binds to this third surface (PDB: 5JK7)^23^. We, therefore, tested whether the binary Vpr/hHR23A complex can interact with UNG2. Biochemical experiments, including size exclusion column chromatography, revealed that a ternary UNG2/Vpr/hHR23A complex can indeed be formed (Fig. 5f–k). The UNG2 association with hHR23 is strictly Vpr dependent, since, in the absence of Vpr, the mixture of hHR23A and UNG2 elutes as two individual peaks (Fig. 5i, k). A structural model for such a complex is shown in Fig. 5l. Whether the UNG2/Vpr/hHR23A complex forms in HIV-1-infected cells and plays a biological role remains to be determined.

## Discussion

HIV-1 Vpr, a small adaptor protein, is the prototype of a large family of primate lentiviral accessory virulence factors. It has been extensively studied over recent years, yet its mode of interaction with target proteins remains poorly understood. The structure of the HIV-1 Vpr/hHR23A complex reported here sheds new light on the molecular mechanisms that underlie the remarkable versatility of this adapter protein and its ability to engage in specific interactions with a large number of structurally unrelated protein partners. Our findings also provide new insights into the cellular mechanisms that Vpr is capable of targeting.

The core three helix bundle structure of Vpr provides different sides/interfaces to engage interacting proteins, while the unstructured N- and C-terminal regions potentially provide additional interaction motifs. Previous studies of HIV-1 Vpr complexes with DCAF1 and post-replication DNA repair proteins targeted by Vpr revealed two distinct Vpr interfaces: a DCAF1-binding interface and a substrate recruitment interface^20,23^ — the latter interacting with multiple targets including UNG2, MUS81 and HLTF^17,20,23^. Strikingly, the current structure of the Vpr/hHR23A complex reveals that hHR23 binds a surface on Vpr that largely overlaps with that engaged by DCAF1. This is remarkable, since hHR23A_223–363_ and DCAF1_1045–1396_ exhibit entirely different three-dimensional structures (Fig. 5m and Supplementary Fig. 5a, b). The ability of Vpr to use the same surface to interact with two structurally unrelated partners, such as hHR23A and DCAF1, is unusual, possibly indicating that other proteins may potentially be engaged.

Both hHR23A and DCAF1 bind the solvent exposed sides of Vpr’s α3 helix, with only a few contacts that are specific to either UBA2 of hHR23A, such as N41 located in the α2 helix and I74 and R77 in the α3 helix, or to DCAF1, such as Vpr’s N-terminal region (Fig. 5n). These divergent, but important contacts probably contribute to the slightly higher affinity of DCAF1_1045–1396_ for Vpr, compared to that of hHR23A_FL_. A similar strategy is evident in Vpr’s recruitment of substrates: Vpr utilizes both common and distinctive interfaces to recruit different post-replication DNA repair proteins to the CRL4^DCAF1^ E3 ligase for ubiquitin-dependent proteasomal degradation^17,20,23^. This structural adaptation may also be important in the specific recruitment of a multitude of additional substrate proteins tentatively identified by recent proteomic studies^45^. Together, these findings illustrate the remarkable versatility of HIV-1Vpr and reveal how it can interact specifically with a wide array of structurally dissimilar cellular targets.

Key residues involved in hHR23A binding are conserved in all HIV-1 Vpr isolates, yet absent in HIV-2 Vpr isolates (Sequences in the Los Alamos Data base; Supplementary Fig. 5e). This suggests that HIV-1 Vpr is uniquely tailored to engage hHR23A, consistent with a previous report^46^. It also contrasts with the observation that the DCAF1 interface residues are entirely conserved among HIV-1 and HIV-2 Vpr proteins. Since hijacking of CRL4^DCAF1^ E3 does not fully account for all known cellular effects of HIV-1 Vpr^25,26,46,47^, its interactions with hHR23A(B) are likely biologically relevant. Considering that Vpr can interact with both hHR23A and hHR23B, and both proteins appear to have largely overlapping functions, binding of Vpr may impact downstream pathways associated with both isoforms.

It is evident from our biochemical studies that Vpr associates with the XPC/hHR23A complex that plays key roles in post-replication DNA repair as well as transcription. Since Vpr binding to hHR23 does not require the presence of XPC in the complex, hHR23 probably acts as a bridge, linking Vpr with XPC. Although the atomic structure of the hHR23A/XPC complex, and in particular the detailed interaction between the XPCB domain of hHR23A and XPC, has not been elucidated yet, this possibility is consistent with predictions from the crystal structure of the homologous yeast Rad23/Rad4 complex^48^. In this structure, the UBA2 domain of Rad23 makes only a few contacts with Rad4, leaving most of the UBA2 domain available for interactions with other proteins, e.g, Vpr (Supplementary Figs. 6 and 7). Although the relative orientation between the domains varies considerably, given the long flexible linker between them, it is nonetheless possible to achieve a satisfactory superposition without steric clashes by rotating the XPCB domain (Supplementary Figs. 6 and 7). Thus, both the homologous human and yeast complexes appear to share similar overall architecture that is predicted to allow for Vpr binding to hHR23A in the presence of XPC.

Although the exact role of the Vpr interaction with the hHR23/XPC complex in HIV-1 biology is not yet known, possible scenarios are suggested by known functions of hHR23/XPC complexes and those of HIV-1 Vpr. In particular, the hHR23/XPC complex recognizes lesions that thermodynamically destabilize DNA duplexes in the early steps of post-replication nucleotide excision repair, including lesions resulting from oxidative DNA damage^29^. HIV-1 reverse transcriptase is error prone, and Vpr has been shown to modulate HIV-1 mutation rates as well as other functional aspects of cellular post-replication DNA repair machinery^49,50^. The latter includes antagonizing select enzymes that mediate processing of branched DNA structures as well as BER^4,15,17,21,51^. This antagonism, mediated by Vpr interacting with the CRL4^DCAF1^ E3 ligase, relieves restrictions imposed by these enzymes on HIV-1 replication^4,22,52^. In contrast, Vpr’s interaction with hHR23/XPC does not involve DCAF1 and does not lead to its ubiquitination and degradation. Thus, it may well be that Vpr recruits the hHR23/XPC complex to facilitate some aspect(s) of proviral DNA repair. Intriguingly, our tentative conclusion that the interaction with hHR23 is specific to HIV-1 Vpr fits well with the previous finding of a more robust remodeling of the cellular DNA repair machinery by HIV-1, compared to HIV-2 Vpr^19^. This is also consistent with the model in which Vpr aids HIV-1 replication by modulating the DNA repair function of the hHR23/XPC complex. Of note, whereas HIV-1 Vpr is known to activate DNA damage checkpoint leading to cell cycle arrest in G2 phase, the Vpr–hHR23 interaction is not required for this function (Supplementary Figs. 8 and  9) consistent with previous reports^25,26,46^.

HIV-1 Vpr activates expression of HIV-1 both prior to integration and post integration. It, therefore, may also modulate expression of cellular genes^53–55^. Interestingly, hHR23/XPC is involved in transcriptional co-activation via histone acetylation and chromatin activation at a subset of RNA polymerase II promoters^31,32^, thus suggesting another role for the Vpr/hHR23 interaction. It is conceivable that the recruitment of the hHR23/XPC complex to active chromatin plays a role in transcriptional effects exerted by HIV-1 Vpr. Future studies are needed to address any possible involvement of the hHR23/XPC complex in the modulation of gene expression and cellular DNA repair processes by HIV-1 Vpr.

In summary, this study and previous structural and biochemical work suggest varied ways by which HIV-1 Vpr can coordinate specific interactions with a large number of structurally unrelated proteins. All lentiviral Vpr family proteins share their core structure with HIV-1 Vpr, yet exhibit extensive amino acid sequence variation on their surfaces. This sequence variability in conjunction with the molecular organization into a plastic three-helix bundle permits promiscuity in protein–protein interactions, providing a versatile means to interfere with multiple key cellular pathways.

## Methods

### Cloning and construction of plasmids

DNA coding for hHR23A_223–363_ and Vpr_1–79_–L-hHR23A_223–363_ was inserted into pET21 (EMD Chemicals). hHR23A_223–363_ and NusA-Vpr_1–79_ gene constructs were inserted into a pET-DUET vector (EMD Chemicals) for co-expression. The Vpr_1–79_–L-hHR23A_223–363_ construct contained a His_6_ tag (LEHHHHHH) at its C-terminus. For mammalian cell expression, cDNAs encoding epitope-tagged full-length (FL) hHR23A, hHR23B, XPC and HIV-1 NL4-3 Vpr wild type and mutant alleles were cloned into pCG vector^56^. All primers used are listed in Supplementary Table 3.

### Protein expression and purification

hHR23A_223–363_, hHR23A_FL_, Trx-hHR23A_FL_, NusA–Vpr_1–79_, UNG2, Vpr_1–79_–L-hHR23A_223–363_, hHR23A_223–363_–L-Vpr_1–79_, Vpr_1–79_/hHR23A_223–363_, Vpr_1–79_/hHR23A_FL_ and Vpr_FL_/hHR23A_FL_ complexes were expressed or co-expressed in *E. coli* Rosetta 2 (DE3) or Rosetta 2 (DE3) pLysS cells (EMD Chemicals) in the presence of 0.1 mM ZnCl_2_, using 0.5 mM isopropyl 1-thio-β-d-galactopyranoside for induction over 16 h at 18 °C. Uniform ^2^H-, ^15^N-, and ^13^C- labeling of the proteins was achieved using modified minimal medium, containing ^2^H_2_O, ^15^NH_4_Cl and ^13^C_6_-glucose as deuterium, nitrogen and carbon sources, while unlabeled proteins were prepared in Luria-Bertani (LB) medium. Cells were harvested by centrifugation at 6000×*g* and re-suspended in lysis buffer, containing 50 mM sodium phosphate (pH 7.5), 10 mM imidazole and 200 mM NaCl. Cells were lysed using a microfluidizer (Microfluidics, MA) or, for hHR23A_223–363_ and Vpr_1–79_–L-hHR23A_223–363_ samples, by sonication (Sonicator 3000; Misonix, Farmingdale, NY). The lysed cells were centrifuged at 40,000×*g*, and the supernatant was applied to a 5-ml HisTrap (GE healthcare) His_6_-tag affinity column, equilibrated in lysis buffer. Bound proteins were eluted using a linear gradient of 0–0.5 M imidazole. Protein containing fractions were further purified over a HiPrep Superdex 75 or HiPrep Superdex 200 (2.6 cm × 60 cm, GE healthcare) gel filtration column, equilibrated in 25 mM sodium phosphate buffer (pH 7.5), 100 mM NaCl, 2 mM DTT and 0.02% sodium azide. Vpr_1–79_–L-hHR23A_223–363_, Vpr_1–79_/hHR23A_223–363_, Vpr_1–79_/hHR23A_FL_ and Vpr_FL_/hHR23A_FL_ complexes were further purified over a HiTrap Q column, equilibrated in 25 mM sodium phosphate buffer (pH 7.5), 2 mM DTT and 0.02% sodium azide. Bound proteins were eluted using a linear gradient of 0–1 M NaCl. Protein fractions were pooled, concentrated for crystallization or buffer-exchanged in Amicon Ultra concentrators (Millipore) for NMR studies against NMR buffers: 10 mM MES (pH 6.5), 10 mM HEPES (pH 7.2 or 7.5) or 25 mM sodium phosphate (pH 7.2 or 7.5), each containing 50 mM NaCl, 2–5 mM DTT or 1 mM TCEP, 0.1 mM ZnSO_4_, 0.1 mM EDTA and 0.02% sodium azide. The correct molecular masses of proteins were confirmed by LC–ESI–TOF mass spectrometry (Bruker Daltonics, Billerica, MA). DCAF_1157–1396_ was expressed in sf9 insect cells and was purified using a 5 ml HisTrap (GE healthcare) and HiPrep Superdex200 (2.6 cm × 60 cm, GE healthcare) gel filtration column as described above.

### Crystallization, structure determination and refinement

Crystals for Vpr_1–79_–L-hHR23A_223–363_ were grown at 16 °C by hanging drop vapor diffusion, using a mixture of 0.5 μl protein (25 mg/ml) with 0.5 μl crystallization buffer (0.1 M Tris, pH 7.5, 10% PEG 4000). Initial crystals were improved by streak seeding. Crystals were cryoprotected by the addition of 35% (v/v) glycerol. Crystals belonging to space group P3_1_21, diffracting to 2.2 Å, were used for structure determination. Data were collected at the 9-2 SSRL (wavelength 0.979 Å, temperature 100 K). Diffraction data were processed, integrated and scaled using XDS^57^. The structure was solved by de novo Zn^2+^ single-wavelength anomalous dispersion (Zn-SAD) phasing. Models were refined using the program BUSTER^41^, followed by several cycles of manual model building and *B* factor sharpening in Coot^43^. Crystallographic statistics are summarized in Table 1.

### NMR spectroscopy

NMR data were collected on Bruker 600, 700, 800, and 900 MHz AVANCE spectrometers. All spectrometers were equipped with *z*-axis gradient, triple resonance cryoprobes. Experiments were performed at 298 K. A homonuclear 2D NOESY spectrum was acquired on unlabeled Vpr_1–79_–L-hHR23A_223–363_. Heteronuclear 2D ^1^H–^15^N HSQC and ^1^H–^13^C HSQC and 3D HNCACB, HN(CO)CACB, HNCA, HN(CO)CA, HBHACONH, HCCCONH, CCCONH, HCCH-TOCSY (mixing time 10.9 ms) and simultaneously ^13^C/^15^N-edited NOESY (mixing time 100 ms) experiments were performed using either 15N- or 15N,13C-labeled Vpr1–79–L-hHR23A223–363, hHR23A223–363-L-Vpr1–79 (only 2D 1H–15N HSQC) and hHR23A223–363 samples. For ^2^H,^15^N,^13^C-labeled Vpr_1–79_–L-hHR23A_223–363_, 3D TROSY-type HNCACB, HN(CO)CACB spectra were also recorded. All data were processed with NMRPipe^58^ and TopSpin 3.1 (Bruker), and analyzed with CCPN^59^.

### NMR structure determination

Structures were calculated in XPLOR-NIH^60,61^. An iterative approach with extensive manual cross-checking of all distance restraints against the NOESY data and the initial structures was employed using CCPN^59^. The final number of NOE-derived distance restraints is 7650, of which 338 are intermolecular (81 Vpr_1–79_/XPCB domain and 257 Vpr_1–79_/UBA2 domain), supplemented by 330 ϕ and ψ backbone torsion angles from TALOS^62^. In addition, a Zn^2+^ ion with tetrahedral coordination to the H33, H71, C76 and H78 side chains of Vpr_1–79_ was added at a late stage in the calculations, using distances from the X-ray structure of the Vpr_1–79_/UBA2 domain. In total, 960 structures were generated, and the 55 lowest energy structures were selected and analyzed using XPLOR-NIH^63^ and PROCHECK-NMR^64^. All structure figures except Supplementary Fig. 1e were generated with Pymol. Supplementary Fig. 1e was generated with MOLMOL^65^.

### In-gel trypsin digestion for mass spectrometry

Gel bands containing (1) a Vpr_1–79_–L-hHR23A_223–363_ solution employed to set up crystallization trays (control) or (2) dissolved crystals of Vpr_1–79_–L-hHR23A_223–363_ were excised and proteins were digested with trypsin^66^. Briefly, gel bands were diced into small pieces (<1 mm^3^) and washed with a solution of 50% acetonitrile (ACN)/25 mM ammonium bicarbonate until no more visible stain was present. The gel pieces were then dehydrated with 100% ACN, reduced with 10 mM DTT at 56 °C for 1 h, followed by alkylation with 55 mM Iodoacetamide (IAA) at room temperature for 45 min in the dark. Excess DTT and IAA were removed by washing the gel pieces with 25 mM ammonium bicarbonate and then twice with 100% ACN. A solution containing 20 ng/µL sequencing grade modified trypsin (Promega Corporation, Madison, WI; catalog#V511A) and 25 mM ammonium bicarbonate was added to cover the gel pieces and digestion was carried out overnight at 37 °C. The resultant tryptic peptides were extracted from the gel with 70% ACN/5% formic acid (FA), vacuum dried, and reconstituted in 18 µL 0.1% FA for nanoflow liquid-chromatography tandem mass spectrometry (nLC–MS/MS analysis).

### Tandem mass spectrometry

Tryptic peptides were analyzed by nLC–MS/MS using a NanoAcquity UPLC (Waters’ Corporation, Milford, MA) interfaced to a Velos Pro linear ion trap mass spectrometer (Thermo Fisher Scientific, Waltham, MA, USA). For each analysis, a 1 µL volume of protein digest was injected onto a C18 column (PicoChip™column packed with Reprosil C18 3 μm 120 Å chromatography media in a 10.5 cm long, 75 μm ID column with a 15 μm tip, New Objective, Inc., Woburn, MA, USA) and eluted off to the mass spectrometer using a 37-min linear gradient of 3–35% ACN/0.1% FA at a flow rate of 300 nL/min. The Velos Pro was operated in positive ionization mode with a spray voltage of 1.95 kV and capillary temperature of 275 °C. Acquisition consisted of cycles of one full-scan MS1 (AGC of 3 × 10^4^, 75 ms maximum ion accumulation time, and *m*/*z* range of 375–1800) followed by eight MS/MS spectra recorded sequentially for the most abundant ions in the ion trap (minimum signal required 1000 counts, 1 × 10^4^ AGC target, 100 ms maximum injection time, isolation width 2*m/z*, normalized collision energy 35, and activation time 10 ms). Dynamic exclusion (30 s) was enabled to minimize redundant selection of peptides previously selected for MS/MS.

### Mass spectrometry data analysis

Collected MS/MS spectra were searched using the MASCOT search engine v2.4.0 (Matrix Science Ltd., London, England)^67^ against the UniProt *E. coli* database, downloaded on September 5, 2014, with 5803 entries, plus the sequence for 23A2V, using the following modifications: static modification of cysteine carbamidomethylation (+57), variable modifications of methionine oxidation (+16), and protein N-terminal acetylation (+42). The mass tolerance was set to 1.4 Da for the precursor ions and 0.8 Da for the fragment ions. Peptide identifications were filtered using the PeptideProphet^TM^ and ProteinProphet^®^ algorithms with a protein threshold cutoff of 99%, minimum of 2 peptides, and peptide threshold cutofff of 90% implemented in Scaffold^TM^ v4.11.0 (Proteome software, Portland, OR).

### GST pulldown assays

1000 pmol of GST-tagged WT hHR23A/B or mutant proteins were incubated with 1000 pmol NusA–Vpr_FL_ in 500 μl PBS buffer (binding buffer) for 30 min at 4 °C. TEV cleavage (50 pmol) was carried out at 4 °C overnight. After centrifugation at 21,000×*g* for 20 min (to remove precipitated, excess Vpr) Glutathion-Sepharose 4B beads (50 μl of a 50% slurry) were added to the supernatant for 1 h at 4 °C. The beads were washed four times with 1 ml of binding buffer, followed by addition of 50 μl SDS–PAGE loading buffer. Proteins were separated by 4–20% SDS–PAGE, transferred to PVDF membrane, and detected by immunoblotting with an anti-His antibody (1:1000 dilution, cat. #05-949, clone His.H8, Millipore).

### Transfections, immunoprecipitations and immunoblotting

Transfections of HEK 293T cells were performed using the calcium phosphate co-precipitation method^68^. Whole-cell extracts were immunoprecipitated with FLAG-M2 beads (Sigma-Aldrich). Immune complexes were eluted by competition with FLAG-peptide under native conditions, separated by SDS–PAGE and transferred to PVDF membranes for immunoblotting. Proteins were detected with antibodies specific for hHR23B (1:1000 dilution, cat. #13525, clone D4W7F, Cell Signaling Technology), FLAG- (1:1000, cat. #F1804, clone M2, Sigma), HA- (1:2000, 12CA5, PMID: 6204768) or myc- (1:1000, 9E10, PMID: 3915782) epitope tags, immune complexes revealed with fluorescent secondary antibodies specific for the mouse (1:10,000, IRDye 800CW Gt anti-mouse IgG Li-Cor cat. #926-322100, or rabbit IgG (IRDye 680RD Gt anti-rabbit IgG 1:10,000, Li-Cor cat. #926-68071) and fluorescent signals quantified using Li-Cor Odyssey imager and software version 3.0.30 (Li-Cor).

### Cell cycle analysis

HEK293T cells were seeded in 12-well plates and co-transfected with plasmids expressing HIV-1 FLAG-tagged HIV-1 Vpr.wt or mutant proteins and GFP marker protein. 48 h post transfection, the cells were trypsinized, washed twice with PBS, fixed with 70% ethanol overnight, washed twice with PBS and stained with propidium iodide. GFP and propidium iodide fluorescence of the cells was analyzed by flow cytometry (BD LSRII). Data were analyzed using FlowJo software (Tree Star Inc., Ashland, OR). One-way ANOVA analysis was performed within GraphPad Prism 9.

### Analytical gel filtration column chromatography

One hundred μl of proteins at 20 μM (hHR23A_FL_, Vpr_FL_/hHR23A_FL_ complex, UNG2, an equimolar mixture of hH23A_FL_ and UNG2 or Vpr_FL_/hHR23A_FL_ complex and UNG2) or 60 μl of proteins at 21 μM (DCAF1_1057–1396_, hHR23A_FL_, Vpr_1–79_/hHR23A_FL_ complex or an equimolar mixture of Vpr_1–79_/hHR23A_FL_ complex and DCAF1_1057–1396_) was injected onto a 24-ml analytical Superdex75 column (equilibrated in 25 mM sodium phosphate, 150 mM NaCl, 5% glycerol, 1 mM DTT and 0.02% NaN_3_) at a flow rate of 0.5 ml/min. Each peak was concentrated and analyzed by SDS–PAGE with Coomassie Blue staining.

### Isothermal titration calorimetry (ITC)

Experiments were performed using a MicroCal PEAQ-ITC calorimeter (Malvern). Proteins were dialyzed overnight against 20 mM sodium phosphate buffer, pH 7.4, 100 mM NaCl and concentrated in an Amicon concentrator (EMD Millipore). NusA–Vpr_1–79_ protein (8.5 µM) was placed in the sample cell and either Trx-hHR23A_FL_ or DCAF1_1045–1396_/DDB1 proteins (100 μM) were added in aliquots at 18 °C. Data analyses were performed with the PEAQ-ITC analysis software.

### Reporting summary

Further information on research design is available in the Nature Research Reporting Summary linked to this article.

## Supplementary information

Supplementary Information

Reporting Summary

## Data Availability

The coordinates of the crystal structure of the Vpr_1-79_/hHR23A-UBA2 complex have been deposited to the Protein Data Bank under accession number PDB 6XQI. The coordinates of the NMR structures of the Vpr_1–79_–L-hHR23A_223–363_ complex have been deposited to the Protein Data Bank under accession number PDB 6XQJ, and the NMR assignments of the complex have been deposited to the Biological Magnetic Resonance Bank (https://bmrb.io/) under entry number 30769, which is available at 10.13018/BMR30769. Previously published PDB accession codes that have been used in this study are 5JK7 (crystal structure of the DDB1/DCAF1/Vpr/UNG2 complex), 2QSF (crystal structure of the yeast Rad23/Rad4 complex), and 4UN2 (crystal structure of the UBA domain of Dsk2 in complex with ubiquitin). Source data are provided with this manuscript.

## Source data

Source Data

## References

[1] Sauter D, Kirchhoff F (2018). Multilayered and versatile inhibition of cellular antiviral factors by HIV and SIV accessory proteins. Cytokine Growth Factor Rev..

[2] Herate C (2016). Uracil DNA glycosylase interacts with the p32 subunit of the replication protein A complex to modulate HIV-1 reverse transcription for optimal virus dissemination. Retrovirology.

[3] Vermeire J (2016). HIV triggers a cGAS-dependent, Vpu- and Vpr-regulated Type I Interferon response in CD4(+) T cells. Cell Rep..

[4] Yan J, Shun MC, Zhang Y, Hao C, Skowronski J (2019). HIV-1 Vpr counteracts HLTF-mediated restriction of HIV-1 infection in T cells. Proc. Natl Acad. Sci. USA.

[5] Connor RI, Chen BK, Choe S, Landau NR (1995). Vpr is required for efficient replication of human immunodeficiency virus type-1 in mononuclear phagocytes. Virology.

[6] Malim MH, Emerman M (2008). HIV-1 accessory proteins-ensuring viral survival in a hostile environment. Cell Host Microbe.

[7] Collins DR, Collins KL (2014). HIV-1 accessory proteins adapt cellular adaptors to facilitate immune evasion. PLoS Pathog..

[8] Tristem M, Purvis A, Quicke DL (1998). Complex evolutionary history of primate lentiviral vpr genes. Virology.

[9] Sharp PM, Bailes E, Stevenson M, Emerman M, Hahn BH (1996). Gene acquisition in HIV and SIV. Nature.

[10] Peeters, M. & Courgnaud, V. Overview of primate lentiviruses and their evolution in non-human primates in Africa. In *HIV Sequence Compendium* (eds Kuiken, C. et al.) 2–23 (Theoretical Biology and Biophysics Group, Los Alamos National Laboratory, Los Alamos, NM, 2002).

[11] Le Rouzic E (2007). HIV1 Vpr arrests the cell cycle by recruiting DCAF1/VprBP, a receptor of the Cul4-DDB1 ubiquitin ligase. Cell Cycle.

[12] Romani B, Cohen EA (2012). Lentivirus Vpr and Vpx accessory proteins usurp the cullin4-DDB1 (DCAF1) E3 ubiquitin ligase. Curr. Opin. Virol..

[13] Hrecka K (2007). Lentiviral Vpr usurps Cul4-DDB1[VprBP] E3 ubiquitin ligase to modulate cell cycle. Proc. Natl Acad. Sci. USA.

[14] Selig L (1997). Uracil DNA glycosylase specifically interacts with Vpr of both human immunodeficiency virus type 1 and simian immunodeficiency virus of sooty mangabeys, but binding does not correlate with cell cycle arrest. J. Virol..

[15] Ahn J (2010). HIV-1 Vpr loads uracil DNA glycosylase-2 onto DCAF1, a substrate recognition subunit of a cullin 4A-ring E3 ubiquitin ligase for proteasome-dependent degradation. J. Biol. Chem..

[16] Laguette N (2014). Premature activation of the SLX4 complex by Vpr promotes G2/M arrest and escape from innate immune sensing. Cell.

[17] Zhou X, DeLucia M, Ahn J (2016). SLX4-SLX1 protein-independent down-regulation of MUS81-EME1 protein by HIV-1 viral protein R (Vpr). J. Biol. Chem..

[18] Lahouassa H (2016). HIV-1 Vpr degrades the HLTF DNA translocase in T cells and macrophages. Proc. Natl Acad. Sci. USA.

[19] Hrecka K (2016). HIV-1 and HIV-2 exhibit divergent interactions with HLTF and UNG2 DNA repair proteins. Proc. Natl Acad. Sci. USA.

[20] Zhou X (2017). HIV-1 Vpr protein directly loads helicase-like transcription factor (HLTF) onto the CRL4-DCAF1 E3 ubiquitin ligase. J. Biol. Chem..

[21] Yan, J. et al. HIV-1 Vpr reprograms CLR4(DCAF1) E3 ubiquitin ligase to antagonize exonuclease 1-mediated restriction of HIV-1 infection. *mBio* **9**, e01732–18 (2018). 10.1128/mBio.01732-18 PMC6199497 30352932

[22] Lv L (2018). Vpr targets TET2 for degradation by CRL4(VprBP) E3 ligase to sustain IL-6 expression and enhance HIV-1 replication. Mol. Cell.

[23] Wu Y (2016). The DDB1-DCAF1-Vpr-UNG2 crystal structure reveals how HIV-1 Vpr steers human UNG2 toward destruction. Nat. Struct. Mol. Biol..

[24] Hansen, E. C. et al. Diverse fates of uracilated HIV-1 DNA during infection of myeloid lineage cells. *Elife* **5**, e18447 (2016). 10.7554/eLife.18447 PMC5030084 27644592

[25] Withers-Ward ES (1997). Human immunodeficiency virus type 1 Vpr interacts with HHR23A, a cellular protein implicated in nucleotide excision DNA repair. J. Virol..

[26] Gragerov A, Kino T, Ilyina-Gragerova G, Chrousos GP, Pavlakis GN (1998). HHR23A, the human homologue of the yeast repair protein RAD23, interacts specifically with Vpr protein and prevents cell cycle arrest but not the transcriptional effects of Vpr. Virology.

[27] Withers-Ward ES, Mueller TD, Chen IS, Feigon J (2000). Biochemical and structural analysis of the interaction between the UBA(2) domain of the DNA repair protein HHR23A and HIV-1 Vpr. Biochemistry.

[28] Yokoi M, Hanaoka F (2017). Two mammalian homologs of yeast Rad23, HR23A and HR23B, as multifunctional proteins. Gene.

[29] Sugasawa K (2019). Mechanism and regulation of DNA damage recognition in mammalian nucleotide excision repair. Enzymes.

[30] Cattoglio C (2015). Functional and mechanistic studies of XPC DNA-repair complex as transcriptional coactivator in embryonic stem cells. Proc. Natl Acad. Sci. USA.

[31] Bidon B (2018). XPC is an RNA polymerase II cofactor recruiting ATAC to promoters by interacting with E2F1. Nat. Commun..

[32] Semer M (2019). DNA repair complex licenses acetylation of H2A.Z.1 by KAT2A during transcription. Nat. Chem. Biol..

[33] Schauber C (1998). Rad23 links DNA repair to the ubiquitin/proteasome pathway. Nature.

[34] Shi, Y. et al. Rpn1 provides adjacent receptor sites for substrate binding and deubiquitination by the proteasome. *Science* **351**, 831–841 (2016). 10.1126/science.aad9421 PMC4980823 26912900

[35] Ortolan TG (2000). The DNA repair protein rad23 is a negative regulator of multi-ubiquitin chain assembly. Nat. Cell Biol..

[36] Morellet N, Bouaziz S, Petitjean P, Roques BP (2003). NMR structure of the HIV-1 regulatory protein VPR. J. Mol. Biol..

[37] Jung J (2014). Binding of HIV-1 Vpr protein to the human homolog of the yeast DNA repair protein RAD23 (hHR23A) requires its xeroderma pigmentosum complementation group C binding (XPCB) domain as well as the ubiquitin-associated 2 (UBA2) domain. J. Biol. Chem..

[38] Dieckmann T (1998). Structure of a human DNA repair protein UBA domain that interacts with HIV-1 Vpr. Nat. Struct. Biol..

[39] Walters KJ, Lech PJ, Goh AM, Wang Q, Howley PM (2003). DNA-repair protein hHR23a alters its protein structure upon binding proteasomal subunit S5a. Proc. Natl Acad. Sci. USA.

[40] Kamionka M, Feigon J (2004). Structure of the XPC binding domain of hHR23A reveals hydrophobic patches for protein interaction. Protein Sci..

[41] Blanc E (2004). Refinement of severely incomplete structures with maximum likelihood in BUSTER-TNT. Acta Crystallogr. D.

[42] Smart OS (2012). Exploiting structure similarity in refinement: automated NCS and target-structure restraints in BUSTER. Acta Crystallogr. D.

[43] Emsley P, Cowtan K (2004). Coot: model-building tools for molecular graphics. Acta Crystallogr. D.

[44] Di Marzio P, Choe S, Ebright M, Knoblauch R, Landau NR (1995). Mutational analysis of cell cycle arrest, nuclear localization and virion packaging of human immunodeficiency virus type 1 Vpr. J. Virol..

[45] Greenwood EJD (2019). Promiscuous targeting of cellular proteins by Vpr drives systems-level proteomic remodeling in HIV-1 infection. Cell Rep..

[46] Mansky LM (2001). Interaction of human immunodeficiency virus type 1 Vpr with the HHR23A DNA repair protein does not correlate with multiple biological functions of Vpr. Virology.

[47] Zhu Q, Wani G, Wani MA, Wani AA (2001). Human homologue of yeast Rad23 protein A interacts with p300/cyclic AMP-responsive element binding (CREB)-binding protein to down-regulate transcriptional activity of p53. Cancer Res..

[48] Min JH, Pavletich NP (2007). Recognition of DNA damage by the Rad4 nucleotide excision repair protein. Nature.

[49] Mansky LM (1996). The mutation rate of human immunodeficiency virus type 1 is influenced by the vpr gene. Virology.

[50] Hu, W. S. & Hughes, S. H. HIV-1 reverse transcription. *Cold Spring Harb. Perspect. Med*. **2**, a006882 (2012). 10.1101/cshperspect.a006882 PMC3475395 23028129

[51] Bouhamdan M (1996). Human immunodeficiency virus type 1 Vpr protein binds to the uracil DNA glycosylase DNA repair enzyme. J. Virol..

[52] Weil AF (2013). Uracil DNA glycosylase initiates degradation of HIV-1 cDNA containing misincorporated dUTP and prevents viral integration. Proc. Natl Acad. Sci. USA.

[53] Poon B, Chen IS (2003). Human immunodeficiency virus type 1 (HIV-1) Vpr enhances expression from unintegrated HIV-1 DNA. J. Virol..

[54] Cohen EA (1990). Identification of HIV-1 vpr product and function. J. Acquir. Immune Defic. Syndr. (1988).

[55] Kino T (1999). The HIV-1 virion-associated protein vpr is a coactivator of the human glucocorticoid receptor. J. Exp. Med..

[56] Tanaka M, Herr W (1990). Differential transcriptional activation by Oct-1 and Oct-2: interdependent activation domains induce Oct-2 phosphorylation. Cell.

[57] Kabsch W (2010). Xds. Acta Crystallogr. D.

[58] Delaglio F (1995). NMRPipe: a multidimensional spectral processing system based on UNIX pipes. J. Biomol. NMR.

[59] Vranken WF (2005). The CCPN data model for NMR spectroscopy: development of a software pipeline. Proteins.

[60] Schwieters CD, Kuszewski JJ, Tjandra N, Clore GM (2003). The Xplor-NIH NMR molecular structure determination package. J. Magn. Reson..

[61] Schwieters CD, Kuszewski JJ, Marius Clore G (2006). Using Xplor–NIH for NMR molecular structure determination. Prog. Nucl. Magn. Reson. Spectrosc..

[62] Cornilescu G, Delaglio F, Bax A (1999). Protein backbone angle restraints from searching a database for chemical shift and sequence homology. J. Biomol. NMR.

[63] Brunger AT (1998). Crystallography & NMR system: a new software suite for macromolecular structure determination. Acta Crystallogr. D.

[64] Laskowski RA, Rullmannn JA, MacArthur MW, Kaptein R, Thornton JM (1996). AQUA and PROCHECK-NMR: programs for checking the quality of protein structures solved by NMR. J. Biomol. NMR.

[65] Koradi R, Billeter M, Wuthrich K (1996). MOLMOL: a program for display and analysis of macromolecular structures. J. Mol. Graph..

[66] Braganza A (2017). UBE3B is a calmodulin-regulated, mitochondrion-associated E3 ubiquitin ligase. J. Biol. Chem..

[67] Perkins DN, Pappin DJ, Creasy DM, Cottrell JS (1999). Probability-based protein identification by searching sequence databases using mass spectrometry data. Electrophoresis.

[68] Graham FL, van der Eb AJ (1973). A new technique for the assay of infectivity of human adenovirus 5 DNA. Virology.

