# Supplementary material for: Structure of HIV-1 Vpr in complex with the human nucleotide excision repair protein hHR23A
Source: Nat Commun. 2021 Nov 25;12:6864. doi: 10.1038/s41467-021-27009-w (PMC8617076; doi:10.1038/s41467-021-27009-w)
Supplement: Supplementary file 1 — Supplementary Information [file 41467_2021_27009_MOESM1_ESM.pdf]

SUPPLEMENTARY INFORMATION

Structure of HIV-1 Vpr in complex with the human nucleotide excision repair protein hHR23A

In-Ja L Byeon[1,2,#], Guillermo Calero[1,2,#*], Ying Wu[1,2], Chang H Byeon[2], Jinwon Jung[2,4], Maria DeLucia[1,2], Xiaohong Zhou[1,2], Simon Weiss [2], Jinwoo Ahn[1,2], Caili Hao[1,3], Jacek Skowronski[1,3], & Angela M Gronenborn[1,2*]

[1]Pittsburgh Center for HIV Protein Interactions, University of Pittsburgh, Pittsburgh, Pennsylvania, USA.

[2]Department of Structural Biology, University of Pittsburgh, Pittsburgh, Pennsylvania, USA.

[3]Department of Molecular Biology and Microbiology, Case Western Reserve School of Medicine, Cleveland, Ohio, USA.

[4]Present address: ABL Bio Inc., 16, Daewangpangyo-ro 712 beon-gil, Bundang-gu, Seongnam-si, Gyeonggi-do, 13488, Rep. of Korea

[#]Theses authors contributed equally: In-Ja L Byeon, Guillermo Calero.

* Correspondence: guc9@pitt.edu (G.C.) and amg100@pitt.edu (A.M.G.)

Supplementary Figures 1-9

Supplementary Tables 1-3

Supplementary References

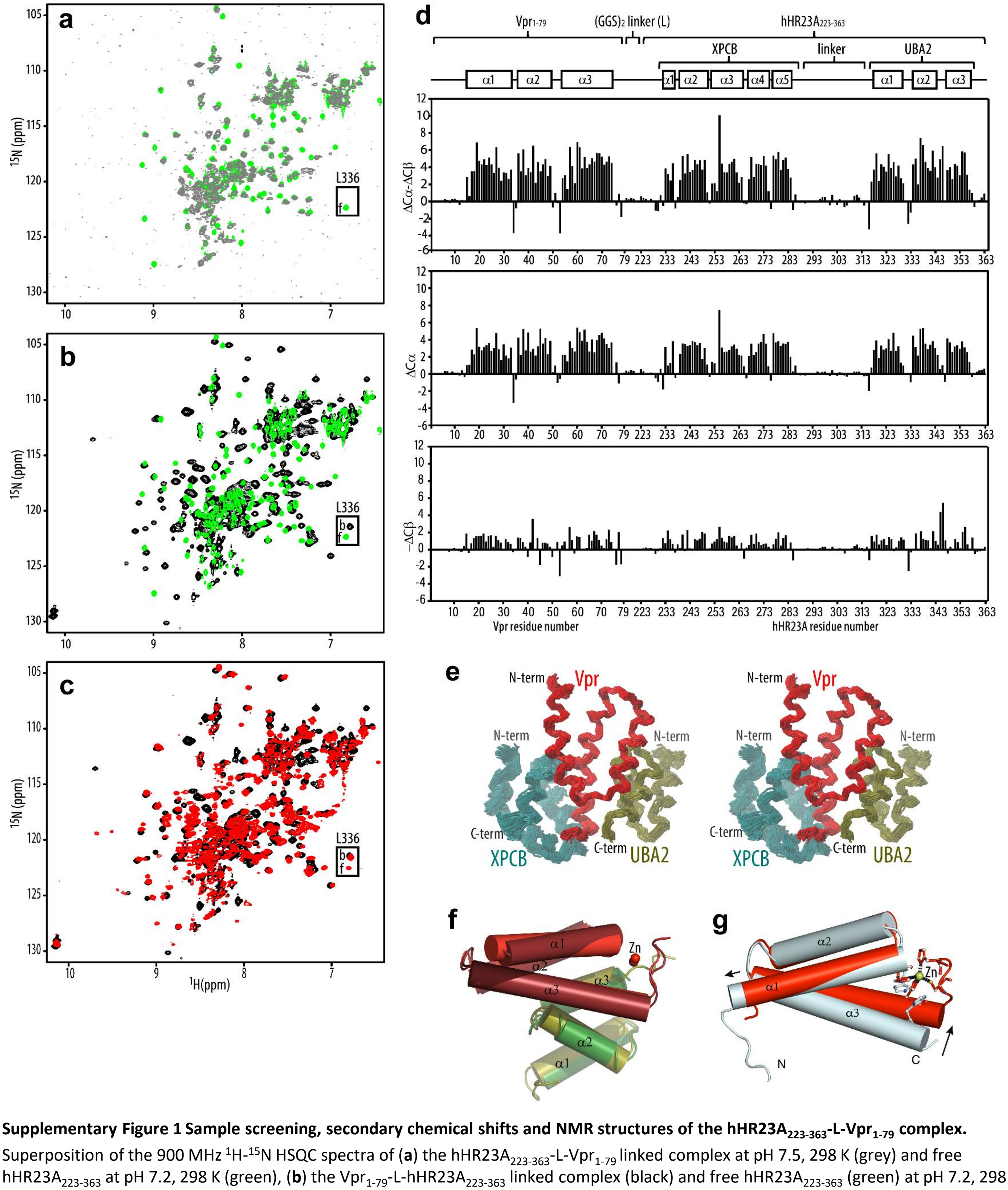

Supplementary Figure 1 Sample screening, secondary chemical shifts and NMR structures of the hHR23A$_{223-363}$-L-Vpr$_{1-79}$ complex.

Superposition of the 900 MHz ^{1}H-^{15}N HSQC spectra of (**a**) the hHR23A$_{223-363}$-L-Vpr$_{1-79}$ linked complex at pH 7.5, 298 K (grey) and free hHR23A$_{223-363}$ at pH 7.2, 298 K (green), (**b**) the Vpr$_{1-79}$-L-hHR23A$_{223-363}$ linked complex (black) and free hHR23A$_{223-363}$ (green) at pH 7.2, 298 K and (**c**) the Vpr$_{1-79}$-L-hHR23A$_{223-363}$ linked complex at pH 7.2, 298 K (black) and the Vpr$_{1-79}$ and hHR23A$_{223-363}$ co-expressed complex at pH 6.5, 291 K (red). Free and Vpr-bound L336 resonances of hHR23A$_{223-363}$ are boxed and labeled. (**d**) Secondary chemical shifts of the Vpr$_{1-79}$-L-hHR23A$_{223-363}$ complex. ΔCα-ΔCβ, ΔCα and -ΔCβ are plotted along the linear amino acid sequence, where ΔCα and ΔCβ are calculated by subtracting random coil values[1] from the Cα and Cβ shifts of each Vpr$_{1-79}$-L-hHR23A$_{223-363}$ residue. (**e**) Stereoview of the final 55 conformer NMR structure ensemble (backbone only; Vpr$_{1-79}$, red; XPCB domain (residues 230-287), teal; UBA2 domain (residues 315-359), olive). (**f**) Superposition of the Vpr (red)/UBA2(olive) part of the NMR structure (lowest energy conformer) of the Vpr$_{1-79}$-L-hHR23A$_{223-363}$ complex and the X-ray structure of the Vpr$_{1-79}$ (ruby)/UBA2 (green) complex in cartoon representation. The Zn^{2+} atoms in the NMR and X-ray structures are depicted as red and ruby spheres, respectively. (**g**) Superposition of hH23A$_{223-363}$-bound Vpr$_{1-79}$ (red, NMR structure) and DCAF1-bound Vpr$_{1-79}$ (grey, X-ray structure; PDB: 5JK7) in cartoon representation, illustrating the difference in relative helix orientations in Vpr (black arrows).

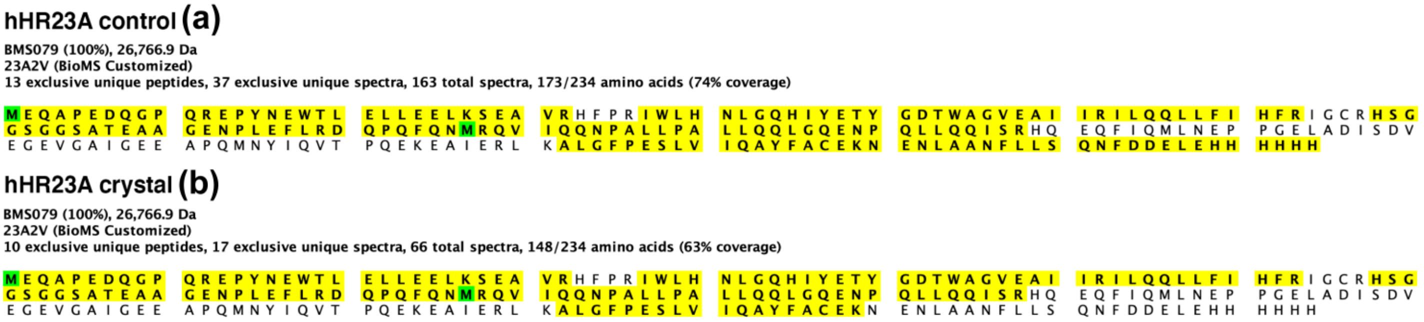

Supplementary Figure 2. **Proteolysis of the C-terminal domain of hHR23A during crystallization identified by tandem mass spectrometry.** (**a**) Sample employed to set up crystallization trays comprised $Vpr_{1\text{-}79}$-L-$hHR23A_{223\text{-}363}$ (control); (**b**) dissolved crystals of $Vpr_{1\text{-}79}$-L-$hHR23A_{223\text{-}363.}$ Peptide sequences identified by mass spectrometry are highlighted in yellow and methionine residues are highlighted in green. Note that the mass spectrometry data show that the dissolved crystal sample is cleaved and missing C-terminal residues (**b**). Source data are provided as a Source Data file.

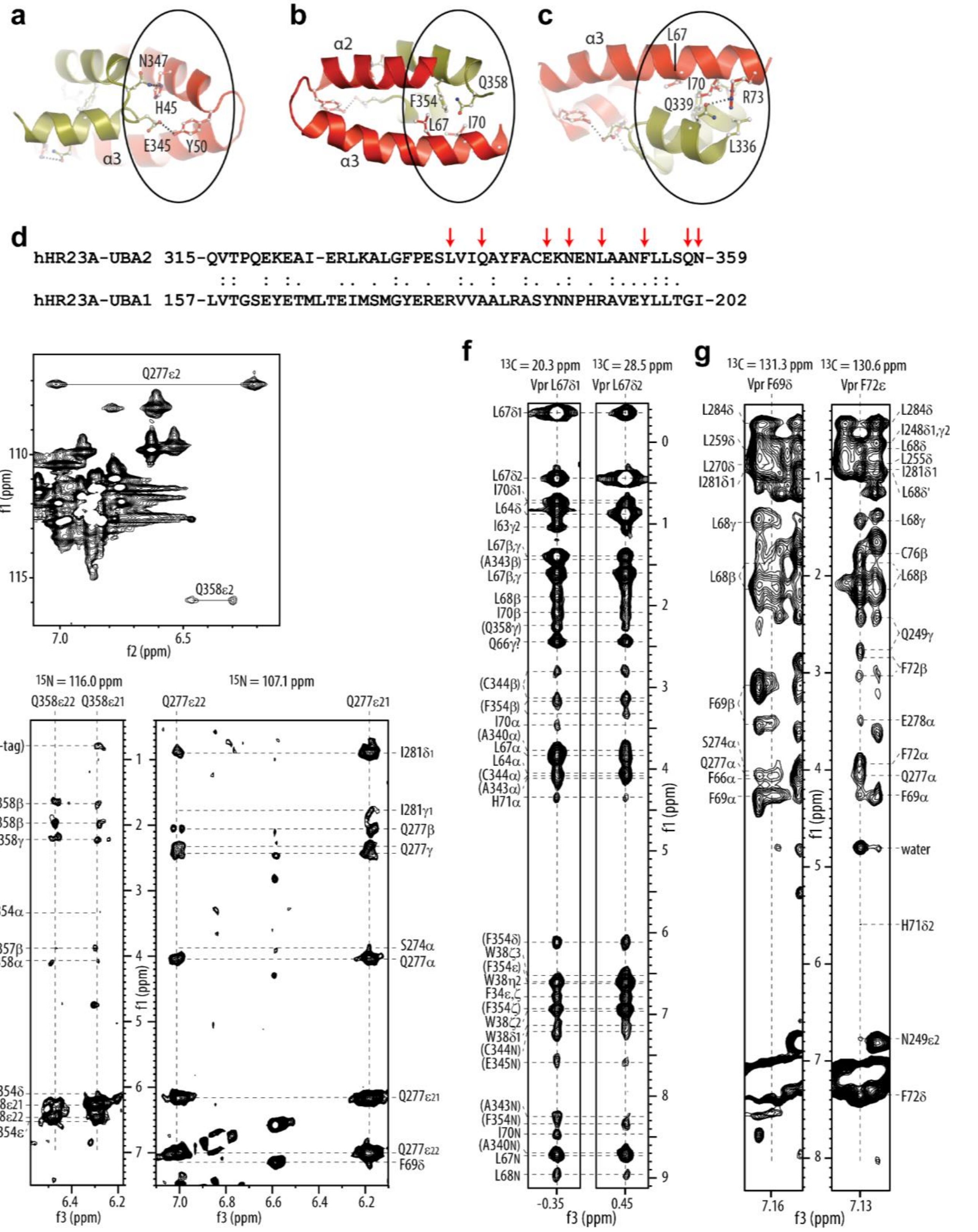

Supplementary Figure 3 Vpr/hHR23A interaction. Close up views of the interacting residues in Vpr (red) and the UBA2 domain (olive) regions. The following amino acid changes were introduced in the current study: E345A/N347A (**a**), F354A/Q358A (**b**) and Q339A (**c**) in UBA2, and H45A (**a**), Y50S (**a**), L67A/I70A (**b,c**) and R73A (**c**) in Vpr. (**d**) Structure-based sequence alignment of hHR23A UBA1 and UBA2 domains. Identical and similar residues are marked with (**:**) and (**.**), respectively. UBA2 residues involved in Vpr binding in the complex (**Fig. 2a-c**) are marked by red arrows. (**e**) Selected regions of the 900 MHz ^{1}H-^{15}N HSQC (**e,** top) and 3D ^{15}N-edited NOESY spectra (**e,** bottom) of the $Vpr_{1\text{-}79}$-L-$hHR23A_{223\text{-}363}$ complex involving the hHR23A Q358 and Q277 $N_{\varepsilon2}H_2$ amino groups. (**f,g**) Selected regions of the 900 MHz 3D ^{13}C-edited NOESY spectrum of the $Vpr_{1\text{-}79}$-L-$hHR23A_{223\text{-}363}$ complex, depicting Vpr L67 methyl group resonances (**f**) and F69 and F72 aromatic resonances (**g**). In (**f**), intermolecular NOEs are indicated by brackets around the resonance label.

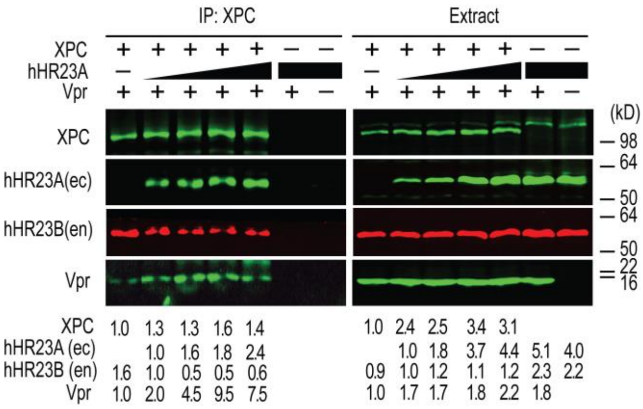

Supplementary Figure 4 Vpr binds the hHR23/XPC complex. This is a biological replicate of the experiment shown in Fig. 4d. FLAG-tagged XPC, HA-tagged hHR23A$_{FL}$ and myc-tagged Vpr$_{FL}$ were expressed alone or in combinations in HEK293 T cells. Cell extracts and XPC immune complexes were analyzed by immunoblotting with antibodies reacting with FLAG-, HA- and myc- epitope tags, and the endogenously expressed hHR23B (en) protein was detected with an antibody specific for the HR23B isoform. Immune complexes were revealed with fluorescent secondary antibodies and fluorescence signals were quantified using a Li-Cor Odyssey imager. Relative levels of the indicated proteins in cell extracts and immune-complexes are shown. Source data are provided as a Source Data file.

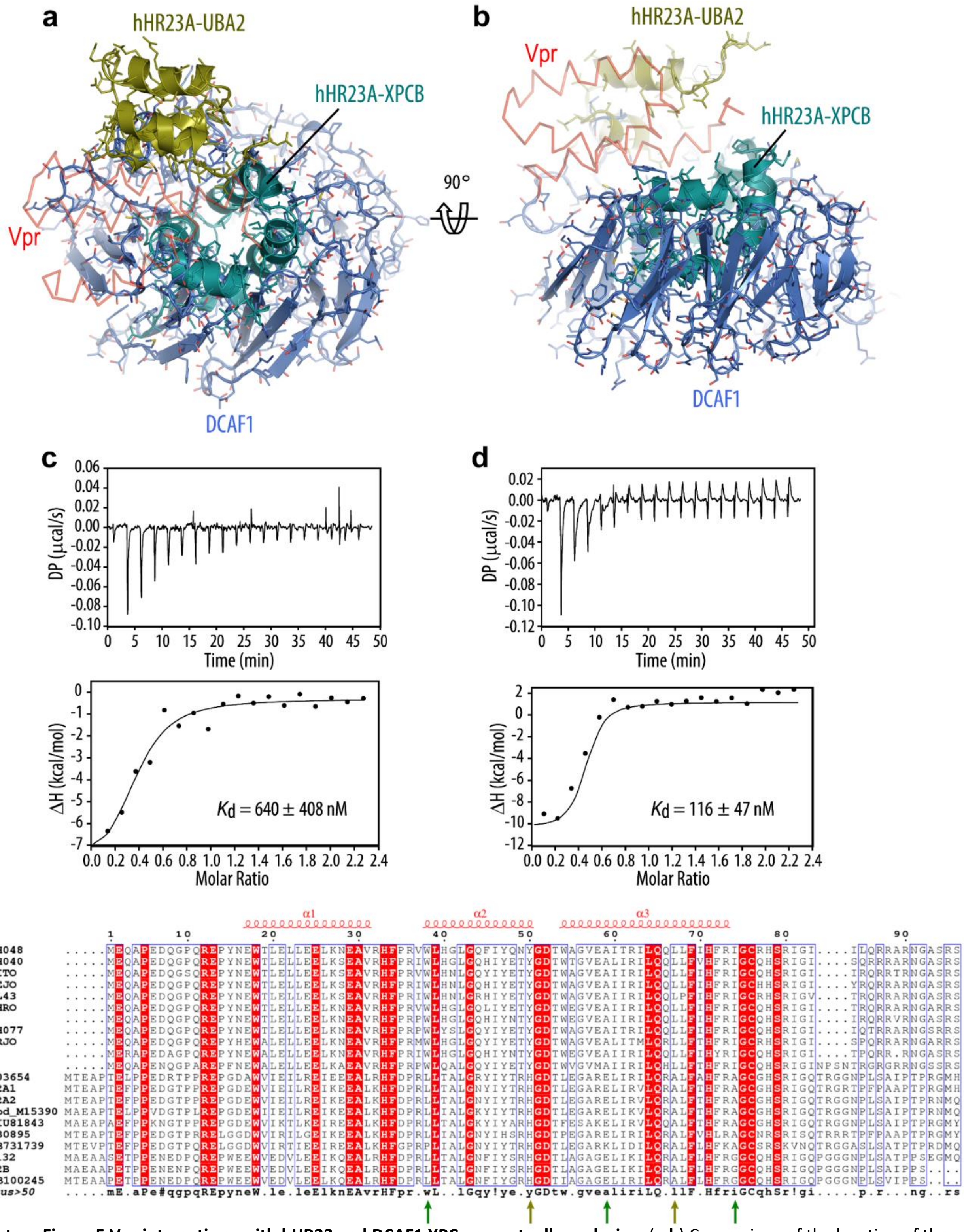

Supplementary Figure 5 Vpr interactions with hHR23 and DCAF1 XPC are mutually exclusive. (**a**,**b**) Comparison of the location of the XPCB and UBA2 domains in the $Vpr_{1\text{-}79}$-L-$hHR23A_{223\text{-}363}$ complex (this study, XPCB domain, teal; UBA2 domain, olive) and $DCAF1_{1045\text{-}1396}$ in the UNG2/$Vpr_{1\text{-}79}$/$DCAF1_{1045\text{-}1396}$/DDB1 (PDB: 5JK7, marine) complex structures. $Vpr_{1\text{-}79}$ is shown as a red backbone trace. The β-sheets loops (WD40 motif of DCAF1) and the turns of the α-helices (helical bundle of the XBCB domain) create equivalent binding surfaces for Vpr's α3 helix. (**c**,**d**) Isothermal titration calorimetry data for NusA-$Vpr_{1\text{-}79}$ interacting with Trx-$hHR23A_{FL}$ (**c**) and $DCAF1_{1045\text{-}1396}$/DDB1(**d**), respectively. Source data are provided as a Source Data file. Two independent experiments were performed yielding similar results. **(e)** Alignment of HIV-1 and HIV-2 Vpr protein sequences. HIV-1 Vpr sequences are of the Vpr used in the present study (NL4-3 Vpr) and those of transmitted founder viruses[2]. HIV-2 Vpr protein sequences were randomly selected from full-length proviral sequences deposited in the Los Alamos HIV sequence database. Consensus HIV-1 (M, N, O) and HIV-2 (H2A1, H2A2, H2B) main group Vpr sequences are also shown in the alignment[3]. Red regions correspond to highly conserved residues, and olive arrows indicate Vpr residues that were mutated in the present study (Y50 and L67) and that were shown to be critical for hHR23A binding. Green arrows indicate HIV-1 Vpr residues that participate in hHR23A binding and are substituted by residues in HIV-2 Vpr that may interfere with hHR23A binding, including HIV-1 W38 (HIV-2 L), A59 (HIV-2 E, potential steric clash) and I74 (HIV-2 A or G). See also Supplementary Table 1.

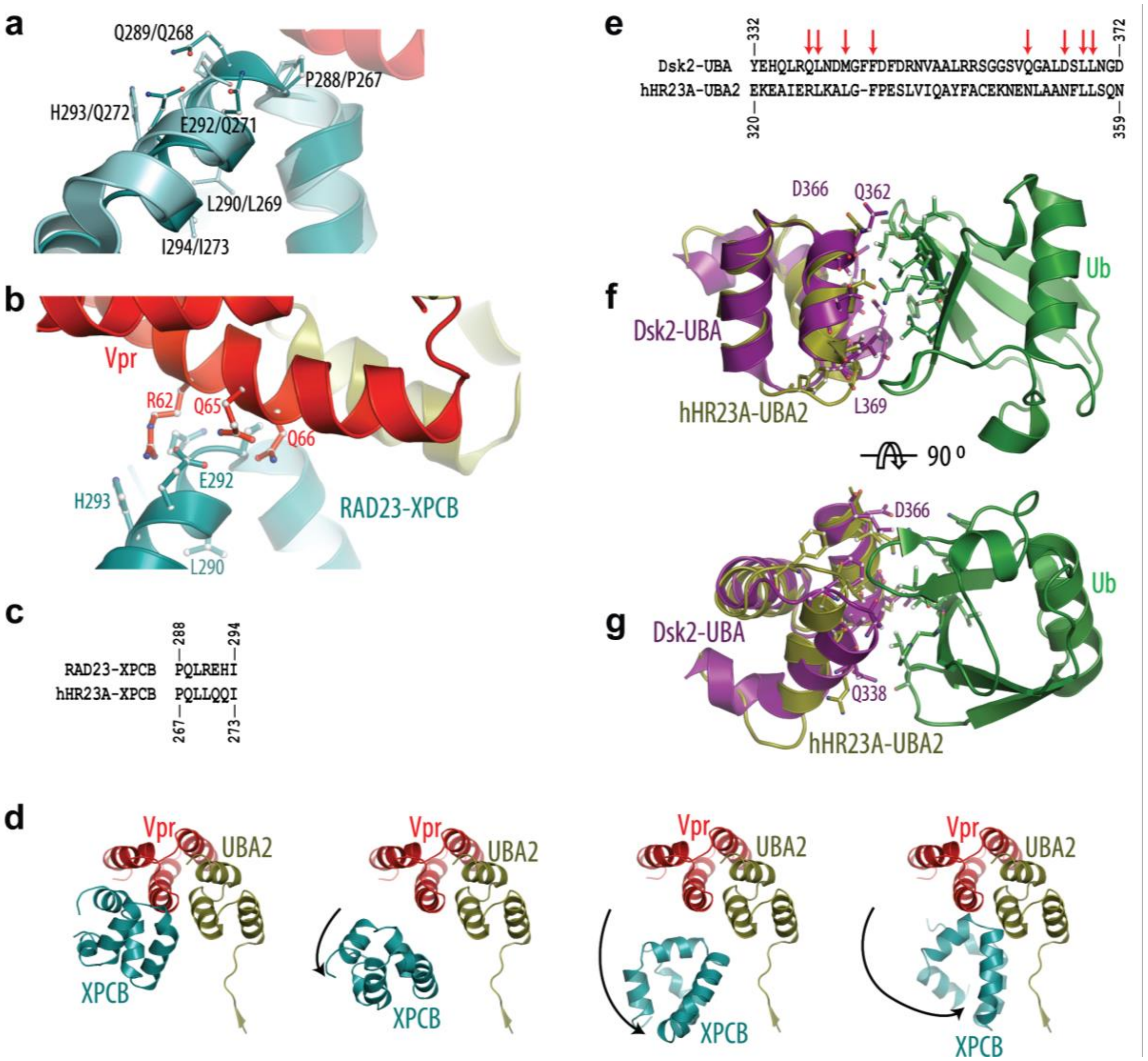

Supplementary Figure 6 Structural comparison of the RAD4/RAD23 and Dsk2-BUA/Ub complexes with Vpr$_{1-79}$-L-hHR23A$_{223-363}$, respectively. (**a**,**b**) Structural alignment (Cα r.m.s.d. of 2.1Å) for the RDB/XPCB domain (light teal) in the RAD4/RAD23 complex and the XPCB domain (teal) in the Vpr$_{1-79}$-L-hHR23A$_{223-363}$ complex. (**c**) Sequence alignment for the small Vpr-binding motif in RAD23 and hHR23A. **(d)** Rotation of the XPCB domain and translocation from its Vpr-bound (left) to Rad4-bound (right) location. Sequence (**e**) and structural alignment (**f**,**g**) for the UBA domain of Dsk2 (purple) and the UBA2 domain of hHR23A (olive) in ribbon representation. Red arrows indicate Dsk2 residues involved in ubiquitin binding (**e**).

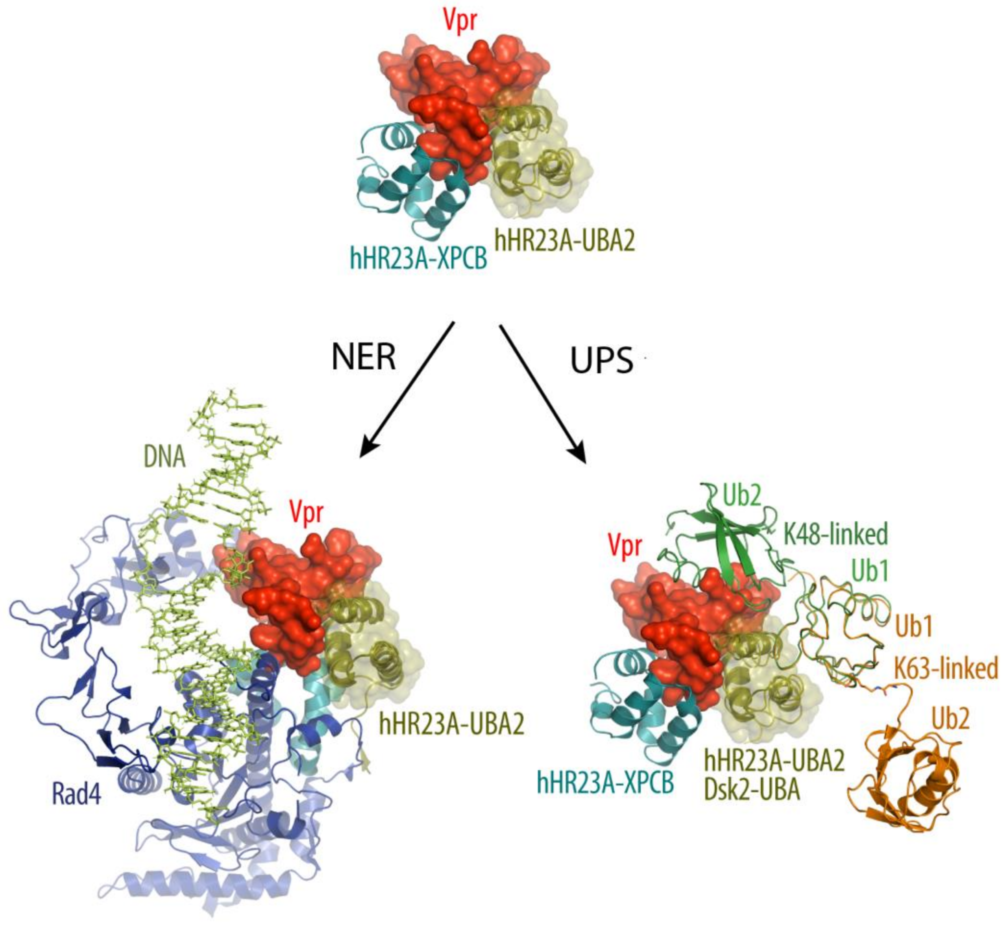

Supplementary Figure 7 Models for the nucleotide excision repair pathway and the ubiquitin proteasome system. Models for the Vpr/hHR23A complex (center) and complexes in the nucleotide excision repair (NER) pathway (left side) and the ubiquitin proteasome system (UPS) (right side). (**left**) Superposition of the UBA2 domain of the Vpr_{1-79}-L-$hHR23A_{223-363}$ complex (current study) and the UBA2 domain of the yeast RAD23/RAD4 bound to a thymidine dimer containing DNA duplex (PDB: 2QSF). In the model, Vpr is located within 7 Å of the thymidine dimer and no steric clashes are observed. Vpr (red) is shown in surface representation, UBA2 of hHR23A in transparent surface and ribbon representation (olive), and the XPCB (teal) and RAD4 (navy) domains are shown in ribbon representations, with the DNA (green) depicted in stick representation. (**right**) Superposition of the UBA2 domain of $hHR23A_{223-363}$ (current study) and the UBA1 domain of the Dsk2/Ubiquitin structure (PDB: 4UN2) as well as K48-linked di-ubiquitin (forest green, PDB: 5GOI) and K63-linked polyubiquitin (orange, PDB: 3HM3). Vpr (red) is shown in surface representation, UBA2 of hHR23A and UBA1 of DSK2 in transparent surface and ribbon representation (olive), with K48-linked ubiquitin (UB1) and K63-linked ubiquitin in ribbon representation (green and orange, respectively).

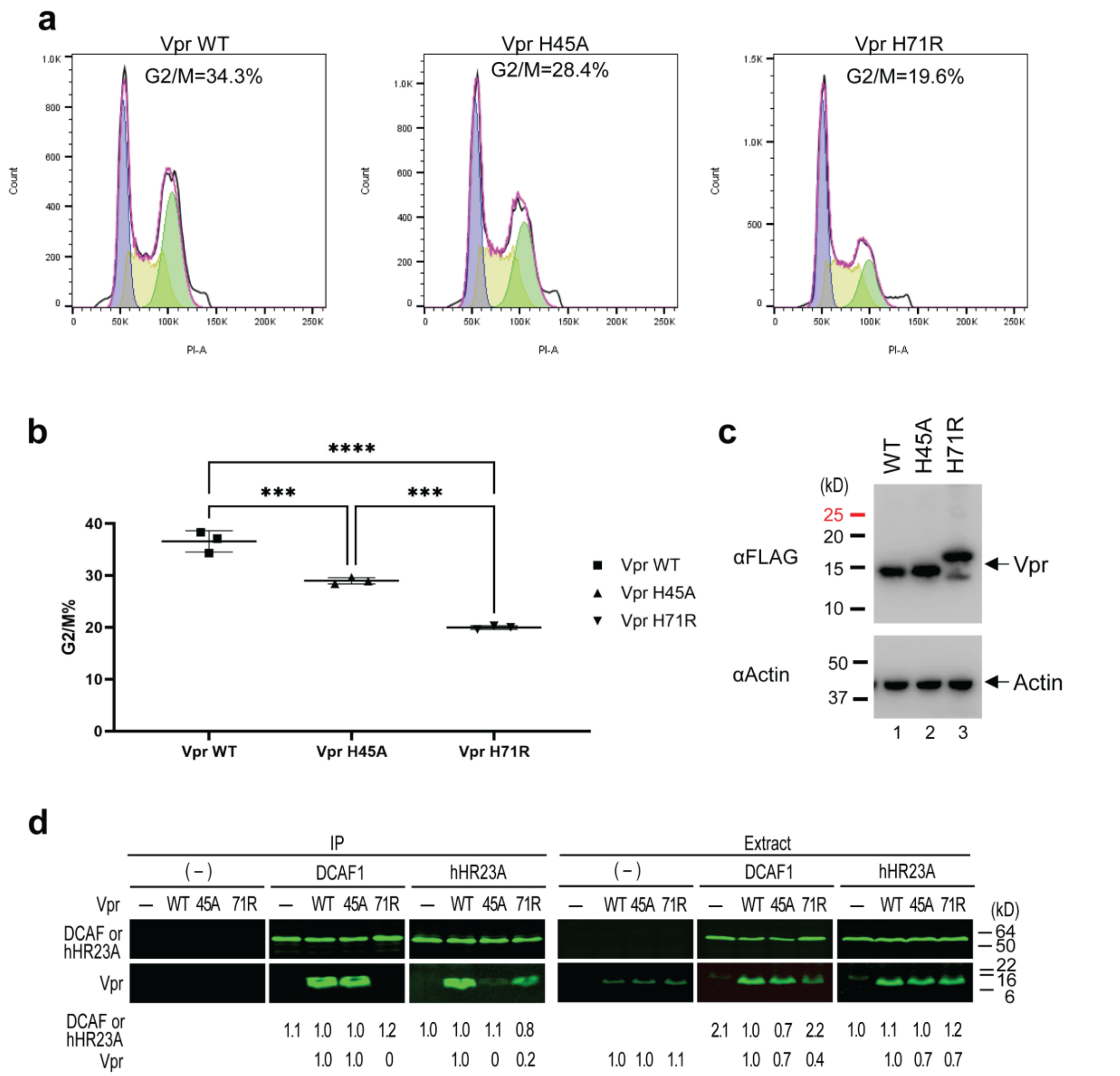

Supplementary Figure 8 Vpr-hHR23 interaction is not required for the induction of the cell cycle arrest in G2 phase. Cell cycle analysis of HEK293T cells transiently co-expressing HIV-1 Vpr WT, H45A or H71R mutant proteins with GFP marker (**a**-**c**). (**a**) Cell cycle profiles in the GFP-positive population with the percentage of G2/M cells indicated. (**b**) Statistical analysis of differences in percentages of cells in G2/M was performed using One-way ANOVA (GraphPad Prism 9). Results from n=3 (triplicate wells) samples over 1 independent experiment are shown. Data are presented as mean values ± SD: Vpr WT, 36.57 ± 2.053; Vpr H45A, 28.97 ± 0.6028; Vpr H71R, 19.97 ± 0.3512. P values: * P<0.05; ** P<0.01; ***P<0.001; ****P<0.0001. The exact P values are: 0.0007 (Vpr WT vs. Vpr H45A), <0.0001 (Vpr WT vs. Vpr H71R) and 0.003 (Vpr H45A vs. Vpr H71R). (**c**) Expression levels of Vpr WT and its mutants were assessed by western blotting. Vpr WT and H45A are FLAG-tagged at the N-terminus and H71R is FLAG and HA tagged at the N-terminus. Two replicates were performed for the experiment yielding similar results. (**d**) The effect of the H45A and H71R mutations in Vpr on hHR23A and DCAF1 binding. FLAG-tagged $DCAF1_{1068\text{-}1389}$ or $hHR23A_{FL}$ complexes were immunoprecipitated, via FLAG-tag, from HEK293T cell extracts transiently co-expressing these proteins and the indicated myc-tagged Vpr variants. The ectopically expressed proteins were revealed by immunoblotting with antibodies specific for the FLAG, and myc epitope tags followed by fluorescent secondary antibodies. Fluorescent signals were quantified using Li-Cor Odyssey imager. Relative levels of the indicated proteins in cell extracts and immune-complexes are shown. Results of one of two replicate experiments performed yielding similar results, are shown. (**b**-**d**) Source data are provided as a Source Data file.

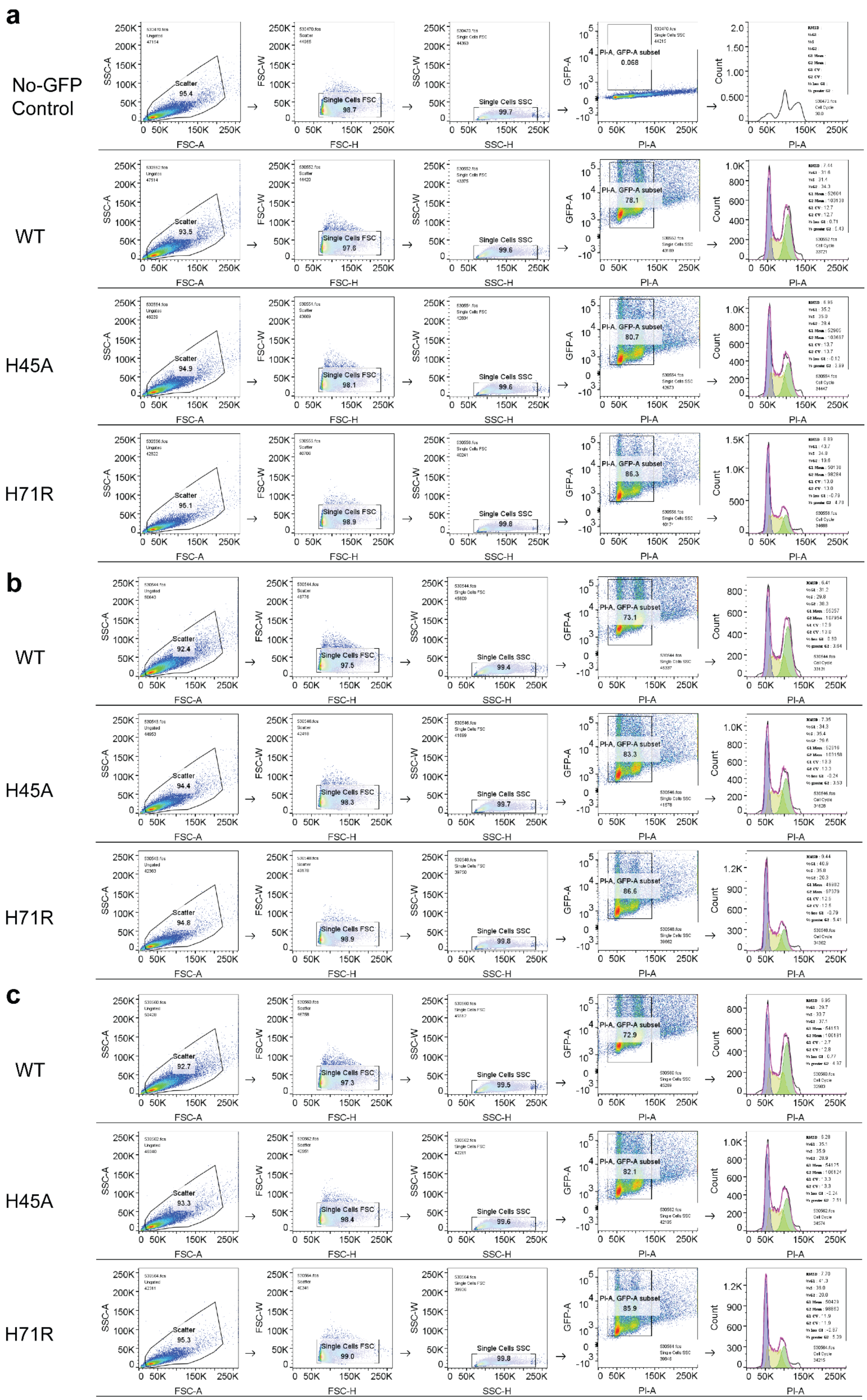

Supplementary Figure 9 Gating strategies used for cell sorting. (a) Gating strategy to sort cells presented on Supplementary Fig. 8a. (a-c) Gating strategy to sort cells presented on Supplementary Fig. 8b.

Supplementary Table 1. Residues exhibiting intermolecular NOEs between Vpr and the UBA2 domain

Vpr residue	UBA2 residue
F34	F354, Q358
W38	L350, F354, N353
N41	N349, L350, N353
L42	L350
H45	E345, N347, L350
I46	E345, L350
Y50	E345
I63	E345, L350
L67	A340, A343, C344, F354, Q358
I70	L336, Q339, A340, A343, F354
H71	N359
R73	Q339
I74	Q358, N359
R77	N359

Supplementary Table 2. Residues exhibiting intermolecular NOEs between Vpr and the XPCB domain

Vpr residue	XPCB residue
L26	E278
E29	E278, I281
Q66	S274
F69	L259, I273, S274, Q277, F280, I281, L284
F72	I248, Q249, P252, L255, I281, L284, N285
C76	P252

Supplementary Table 3. List of all primers used in this study.

Name	Primer sequences
hHR23A.5.Xba	GGAGAATCTAGAatggccgtcaccatc
hHR23A.3.Mlu	GGAGAACGCGTtcactcgtcatcaaagttctg
hHR23B.5.Xba	GGAGAATCTAGAatgcaggtcaccctgaa
hHR23B.3.Mlu	GGAGAACGCGTtcaatcttcatcaaagttctgctg
XPC.5.Spe	GGAGAAACTAGTatggctcggaaacgc
XPC.3.Mlu	GGAGAACGCGTCTAtcacagctgctcaaatgg
NL43.Vpr.5.Nhe	GGAAGAAGCTAGCatggaacaagccccag
NL43.Vpr.3.Mlu	GGAAGAAACGCGTctaggatctactggctcca
NdeI.hHR23A$_{223\text{-}363}$	GATATACATatggccacggaagcagcaggagagaac
hHR23A$_{223\text{-}363}$.XhoI	GTGGTGCTCGAGctcgtcatcaaagttctgactcaggaggaa
Vpr$_{1\text{-}79}$-L-hHR23A$_{223\text{-}363}$	AGAATTGGGTGTCGACATAGCggtggatccggaggctccGCCACGGAAGCAGCAG GAGAG
BamHI.TEV.SalI.hHR23A$_{223\text{-}363}$	CAGCCAGGATCCgcaggaaaacctgtatttccagagtgtcgacgccacggaagcagcaggagag
hHR23A$_{223\text{-}363}$.ST.NotI	CATTATGCGGCCGCtcactcgtcatcaaagttctgactcag
Vpr.NdeI	GATATACATatggaacaagccccagaagac
VPR$_{1\text{-}79}$.STOP.XhoI	GTGGTGCTCGAGtcagctatgtcgacacccaattctgaa

Supplementary References

1. Wishart, D.S. & Sykes, B.D. The 13C chemical-shift index: a simple method for the identification of protein secondary structure using 13C chemical-shift data. *J Biomol NMR* **4**, 171-80 (1994).
2. Ochsenbauer, C. et al. Generation of transmitted/founder HIV-1 infectious molecular clones and characterization of their replication capacity in CD4 T lymphocytes and monocyte-derived macrophages. *J Virol* **86**, 2715-28 (2012).
3. Hrecka, K. et al. HIV-1 and HIV-2 exhibit divergent interactions with HLTF and UNG2 DNA repair proteins. *Proc Natl Acad Sci U S A* **113**, E3921-30 (2016).
