# Supplementary material for: Structure of HIV-1 Vpr in complex with the human nucleotide excision repair protein hHR23A
Source: Nat Commun. 2021 Nov 25;12:6864. doi: 10.1038/s41467-021-27009-w (PMC8617076; doi:10.1038/s41467-021-27009-w)
Supplement: Supplementary file 2 — Reporting Summary [file 41467_2021_27009_MOESM2_ESM.pdf]

nature research

Corresponding author(s): Angela M. Gronenborn

Last updated by author(s): October 22, 2021

Reporting Summary

Nature Research wishes to improve the reproducibility of the work that we publish. This form provides structure for consistency and transparency in reporting. For further information on Nature Research policies, see our Editorial Policies and the Editorial Policy Checklist.

Statistics

For all statistical analyses, confirm that the following items are present in the figure legend, table legend, main text, or Methods section.

n/a	Confirmed	
☐	☒	The exact sample size (*n*) for each experimental group/condition, given as a discrete number and unit of measurement
☒	☐	A statement on whether measurements were taken from distinct samples or whether the same sample was measured repeatedly
☐	☒	The statistical test(s) used AND whether they are one- or two-sided<br>*Only common tests should be described solely by name; describe more complex techniques in the Methods section.*
☒	☐	A description of all covariates tested
☒	☐	A description of any assumptions or corrections, such as tests of normality and adjustment for multiple comparisons
☐	☒	A full description of the statistical parameters including central tendency (e.g. means) or other basic estimates (e.g. regression coefficient) AND variation (e.g. standard deviation) or associated estimates of uncertainty (e.g. confidence intervals)
☐	☒	For null hypothesis testing, the test statistic (e.g. *F*, *t*, *r*) with confidence intervals, effect sizes, degrees of freedom and *P* value noted<br>*Give P values as exact values whenever suitable.*
☒	☐	For Bayesian analysis, information on the choice of priors and Markov chain Monte Carlo settings
☒	☐	For hierarchical and complex designs, identification of the appropriate level for tests and full reporting of outcomes
☒	☐	Estimates of effect sizes (e.g. Cohen's *d*, Pearson's *r*), indicating how they were calculated

Our web collection on statistics for biologists contains articles on many of the points above.

Software and code

Policy information about availability of computer code

Data collection	NMR data were collected with TopSpin version 3.1. ITC data were collected with Microcal PEAK-ITC. Cell cycle data were collected with BD FACSDiva™ software. Western blot fluorescent signals were imaged using Li-Cor Odyssey scanner (verision 3.0.30).
Data analysis	NMR data were processed using NMRpipe version 8.7 and analyzed with TopSpin version 3.1, CCPN version 2.3.1, PROCHECK-NMR version 3.5.4, MOLMOL version 2K.2 and TALOS+ version; NMR structures were determined using XPLOR-NIH version 2.48.<br>X-ray diffraction data were processed, integrated and scaled using XDS version January 26, 2018, models were refined using the program BUSTER version 2.10.3, followed by several cycles of manual model building and B factor sharpening in Coot version 10.6.8.<br>ITC data analysis was performed with PEAK-ITC software version 1.21.<br>Cell cycle data analysis was performed with FlowJo software version 10.<br>Western blot fluorescent signals were quantified using Li-Cor Odyssey scanner software version 3.0.30. Statistical analysis of differences in percentages of cells in G2/M was performed using One-way ANOVA (GraphPad Prism 9).

For manuscripts utilizing custom algorithms or software that are central to the research but not yet described in published literature, software must be made available to editors and reviewers. We strongly encourage code deposition in a community repository (e.g. GitHub). See the Nature Research guidelines for submitting code & software for further information.

Data

Policy information about availability of data

All manuscripts must include a data availability statement. This statement should provide the following information, where applicable:

- Accession codes, unique identifiers, or web links for publicly available datasets
- A list of figures that have associated raw data
- A description of any restrictions on data availability

Accession codes for deposited data: the coordinate of the X-ray structure of the Vpr1-79/hHR23A-UBA2 has been deposited to the Protein Data Bank under accession number PDB 6XQI. The coordinates of the NMR structures of the Vpr1-79-L-hHR23A223-363 complex have been deposited to the Protein Data Bank under accession number PDB 6XQJ, and the NMR assignments of the complex have been deposited to the Biological Magnetic Resonance Bank under entry 30769.

Field-specific reporting

Please select the one below that is the best fit for your research. If you are not sure, read the appropriate sections before making your selection.

☒ Life sciences ☐ Behavioural & social sciences ☐ Ecological, evolutionary & environmental sciences

For a reference copy of the document with all sections, see nature.com/documents/nr-reporting-summary-flat.pdf

Life sciences study design

All studies must disclose on these points even when the disclosure is negative.

Sample size	Regarding NMR data, three different (2H,15N,13C-labeled, 15N,13C-labeled, unlabeled) Vpr1-79-L-hHR23A223-363 NMR samples at two different pH (7.5 and 7.2) values were prepared for NMR structure determinations.<br>Regarding the analyses of Vpr-hHR23- and/or Xpc complex formation upon ectopic expression in HEK293T cells, Vpr mutants used were based on their phenotypic characteristics that were relevant to this study.<br>Regarding the GST-pull down, analytical gel-filtration chromatography and ITC experiments, the proteins and mutants were rationally chosen based on the structures determined in this current study and available in the literature.
Data exclusions	All the NMR data from the NMR samples listed above were used for the analyses without exclusion.<br>No data were excluded from analyses of the GST-pull down, analytical gel-filtration chromatography, ITC and cell cycle arrest experiments.<br>No data were excluded from analyses of Vpr-hHR23- and/or Xpc complex formation upon ectopic expression in HEK293T cells.
Replication	GST-pull down data for hHR23A and Vpr were performed with three replicates for each experiment yielding similar results. The number of replicates is indicated in Figure legends.<br>Analytical gel-filtration chromatography and ITC data were obtained with two independent experiments yielding similar results.<br>Analyses of Vpr-hHR23- and/or Xpc complex formation upon ectopic expression in HEK293T cells were performed in multiple independent experiments; with duplicate biological replicates. The number of biological replicates is indicated in Figure legends. All independent replicates yielded similar results. When applicable, the replicate data were provided in Supplementary Figures.<br>Analyses of cell cycle arrest data were performed in multiple independent experiments. The number of biological replicates is indicated in Figure legends. All independent replicates yielded similar results.
Randomization	NMR samples were prepared and screened by HSQC NMR spectrum for its homogeneity and structural fold so randomization is not applicable.<br>Not applicable to the analyses of Vpr-hHR23- and/or Xpc complex formation upon ectopic expression in HEK293T cells.
Blinding	NMR samples were prepared and screened by HSQC NMR spectrum for its homogeneity and structural fold so randomization is not applicable.<br>Investigators were blinded when possible during quantifications of data from analyses of Vpr-hHR23- and/or Xpc complex formation upon ectopic expression in HEK293T cells.

Reporting for specific materials, systems and methods

We require information from authors about some types of materials, experimental systems and methods used in many studies. Here, indicate whether each material, system or method listed is relevant to your study. If you are not sure if a list item applies to your research, read the appropriate section before selecting a response.

Materials & experimental systems

n/a	Involved in the study
	☒ Antibodies
	☒ Eukaryotic cell lines
☒	☐ Palaeontology and archaeology
☒	☐ Animals and other organisms
☒	☐ Human research participants
☒	☐ Clinical data
☒	☐ Dual use research of concern

Methods

n/a	Involved in the study
☒	☐ ChIP-seq
	☒ Flow cytometry
☒	☐ MRI-based neuroimaging

Antibodies

Antibodies used

Antibody, Supplier, Cat #, Clone name
anti-Flag, Sigma, F1804, M2
anti-HA, Biolegend, 901503, 16B12
anti-HA, PMID: 6204768, 12CA5
anti-myc, PMID: 3915782, 9E10
anti-HR23B Cell Signaling Technology 13525 D4W7F
IRDye 800CW Gt anti-mouse IgG, Li-Cor, 926-322100
IRDye 680RD Gt anti-rabbit IgG, Li-Cor, 926-68071

Validation

According to manufacturer's website the Anti-Flag M2 antibody is used for the detection of Flag fusion proteins. This monoclonal antibody is produced in mouse and recognizes the FLAG sequence at the N-terminus, Met N-terminus, and C-terminus. The antibody is also able to recognize FLAG at an internal site. M2, unlike M1 antibody is not Calcium dependent. F1804 is an affinity purified, FLAG M2 antibody, increasing sensitivity in most applications. Method of purification - Protein A. It is recommended for immunoblotting, immunoprecipitation, immunocytochemistry, immunofluorescence, ELISA, EIA, chromatin immunoprecipitation, electron microscopy, flow cytometry, supershift assays. The anti-Flag M2 mAb was further validated by western blotting with extracts from HEK293T cells that were transiently expressing, or not, recombinant protein tagged with Flag- epitope. The antibody detected protein bands of the expected sizes only in extracts from cells expressing the FLAG, but not other epitope tagged proteins.

The anti-HA 12CA5 mouse monoclonal antibody is reactive with an epitope of the influenza hemagglutinin protein (PMID: 6204768). It has been extensively used as a general epitope tag in expression vectors for immunoblotting and immunoprecipitation experiments. The antibody was purified by affinity chromatography on protein G agarose and validated by western blotting with extracts from HEK293T cells that were transiently expressing, or not, recombinant proteins tagged with HA- epitope. The antibody revealed protein bands of the expected sizes only in extracts from cells expressing the HA- epitope tagged proteins.

The anti-myc 9E10 mouse monoclonal antibody is reactive with an epitope of the human c-myc protein (PMID: 3915782). It has been extensively used as a general epitope tag in expression vectors for immunoblotting and immunoprecipitation experiments. The antibody was purified by affinity chromatography on protein G agarose and validated by western blotting with extracts from HEK293T cells that were transiently expressing, or not, recombinant proteins tagged with myc- epitope. The antibody revealed protein bands of the expected sizes only in extracts from cells expressing the myc- epitope tagged proteins.

According to the manufacturer's website the Rad23B (D4W7F) Rabbit mAb recognizes endogenous levels of total human Rad23B protein in western blotting. This antibody does not cross-react with Rad23A protein. It was produces by immunizing animals with a synthetic peptide corresponding to residues surrounding Ala140 of human Rad23B protein. The anti-HR23B D4W7F monoclonal antibody was further validated by western blotting with extracts from cells transiently over-expressing, or not, human hHR23B protein. This antibody detected elevated levels of a protein that co-migrated with the endogenous reacting band, in cells ectopically over-expressing hHR23B.

According to the manufacturer's website, the IRDye 680RD Gt anti-rabbit IgG, (Li-Cor, 926-68071) antibody was purified by affinity chromatography using pooled rabbit IgG covalently linked to agarose. Based on ELISA and flow cytometry, this antibody reacts with the heavy and light chains of rabbit IgG, and with the light chains of rabbit IgM and IgA. This antibody was tested by dot blot and and/or solid-phase adsorbed for minimal cross-reactivity with human, mouse, rat, sheep, and chicken serum proteins, but may cross-react with immunoglobulins from other species. The conjugate has been specifically tested by the. manufacturer and qualified for Western blot and In-Cell Western™ assay applications. This antibody was was further validated by western blotting and found to specifically reveal only the antigens that were reacted with rabbit, but not mouse IgG, at concentrations used in the presented here studies.

According to the manufacturer's website, the IRDye 800RD Gt IRDye 800CW Gt anti-mouse IgG, (Li-Cor, 926-322100) antibody was purified by affinity chromatography using pooled mouse IgG covalently linked to agarose. Based on ELISA and flow cytometry, this antibody reacts with the heavy and light chains of mouse IgG1, IgG2a, IgG2b, and IgG3, and with the light chains of mouse IgM and IgA. This antibody was tested by dot blot and and/or solid-phase adsorbed for minimal cross-reactivity with human, rabbit, goat, rat, and horse serum proteins, but may cross-react with immunoglobulins from other species. The conjugate has been specifically tested and qualified for Western blot applications. This antibody was was further validated by western blotting and found to specifically reveal only the antigens that were reacted with mouse, but not rabbint IgG, at concentrations used in the presented here studies.

Eukaryotic cell lines

Policy information about cell lines

Cell line source(s)	HEK293 T cells (ATCC cat. no. ACS-4500)
Authentication	By their morphology and ease of transfection
Mycoplasma contamination	Mycoplasma free ~ 15 years ago.
Commonly misidentified lines (See ICLAC register)	No commonly misidentified cell lines were used in the study.

Flow Cytometry

Plots

Confirm that:

- [x] The axis labels state the marker and fluorochrome used (e.g. CD4-FITC).
- [x] The axis scales are clearly visible. Include numbers along axes only for bottom left plot of group (a 'group' is an analysis of identical markers).
- [x] All plots are contour plots with outliers or pseudocolor plots.
- [x] A numerical value for number of cells or percentage (with statistics) is provided.

Methodology

Sample preparation	HEK293T cells were seeded in 12-well plates were co-transfected with plasmids expressing HIV-1 FLAG-tagged HIV-1 Vpr.wt or mutant proteins and GFP marker protein. 48 hours post transfection, the cells were trypsinized, washed twice with PBS, fixed with 70% ethanol overnight, washed twice with PBS and stained with propidium iodide.
Instrument	GFP and propidium iodide fluorescence of the cells was then analyzed flow cytometry (BD LSRII).
Software	Data were analyzed using FlowJo software (Tree Star Inc., Ashland, OR). One-way ANOVA analysis was performed within GraphPad Prism 9.
Cell population abundance	During sample measurements and data collection, initial gate was used to ensure a cell count of 30,000 cells or events was collected for the relevant cell population.
Gating strategy	Initial cell populations were gated using FSC and SSC to remove cell debris and large clumps or aggregates of cells. All samples were then gated by FSC-W/FSC-H and SSC-W/SSC-H gating to select singlet cells. Then GFP positive cells were gated for cell cycle analysis. Control cells (no GFP) were used to set gates. See Supplementary Figure 9 and the Figure legend.

- [x] Tick this box to confirm that a figure exemplifying the gating strategy is provided in the Supplementary Information.
